# Variant Signal Peptides of Vaccine Antigen, FHbp, Impair Processing Affecting Surface Localization and Antibody-Mediated Killing in Most Meningococcal Isolates

**DOI:** 10.3389/fmicb.2019.02847

**Published:** 2019-12-19

**Authors:** Ronni A. G. da Silva, Andrey V. Karlyshev, Neil J. Oldfield, Karl G. Wooldridge, Christopher D. Bayliss, Ali Ryan, Ruth Griffin

**Affiliations:** ^1^Centre for Biomolecular Sciences, University of Nottingham, Nottingham, United Kingdom; ^2^School of Life Sciences, Pharmacy and Chemistry, Kingston University, Kingston upon Thames, United Kingdom; ^3^Department of Genetics and Genome Biology, University of Leicester, Leicester, United Kingdom

**Keywords:** meningoccocus, FHbp, vaccine, signal peptide, lipoprotein, Lnt, Slam

## Abstract

Meningococcal lipoprotein, Factor H binding protein (FHbp), is the sole antigen of the Trumenba vaccine (Pfizer) and one of four antigens of the Bexsero vaccine (GSK) targeting *Neisseria meningitidis* serogroup B isolates. Lipidation of FHbp is assumed to occur for all isolates. We show in the majority of a collection of United Kingdom isolates (1742/1895) non-synonymous single nucleotide polymorphisms (SNPs) in the signal peptide (SP) of FHbp. A single SNP, common to all, alters a polar amino acid that abolishes processing: lipidation and SP cleavage. Whilst some of the FHbp precursor is retained in the cytoplasm due to reduced binding to SecA, remarkably some is translocated and further surface-localized by Slam. Thus we show Slam is not lipoprotein-specific. In a panel of isolates tested, the overall reduced surface localization of the precursor FHbp, compared to isolates with an intact SP, corresponded with decreased susceptibility to antibody-mediated killing. Our findings shed new light on the canonical pathway for lipoprotein processing and translocation of important relevance for lipoprotein-based vaccines in development and in particular for Trumenba.

## Introduction

*Neisseria meningitidis* is a leading cause of bacterial meningitis and sepsis with high fatality (up to 50% when untreated) and high frequency (more than 10%) of severe sequelae (Rappuoli et al., 2018). Polysaccharide-based vaccines are effective in preventing disease caused by isolates of serogroups A, C, W, and Y but are ineffective against those of serogroup B (MenB) (Pace and Pollard, 2007). The lipoprotein, Factor H binding protein (FHbp), is a major virulence factor, which recruits human factor H (fH) to the meningococcal surface preventing complement from binding to the bacterium and thus inhibiting bacteriolysis by the alternative complement pathway (Schneider et al., 2006). The amino acid sequence of FHbp varies with identities as low as 60% between isolates which led to the classification of this lipoprotein into subfamily A (subdivided into variant groups 2 and 3) and subfamily B (variant group 1) (Masignani et al., 2003; Fletcher et al., 2004; Brehony et al., 2009; Jiang et al., 2010). Despite this variation, FHbp emerged as a promising vaccine candidate due to its ability to stimulate a strong serum bactericidal antibody (SBA) response capable of killing diverse group B isolates (Fletcher et al., 2004). It is thought that FHbp-specific antibodies not only promote bactericidal killing by the classical pathway but also via amplification of the alternative pathway, by preventing fH from binding to FHbp (Giuntini et al., 2011).

**FIGURE 1:**
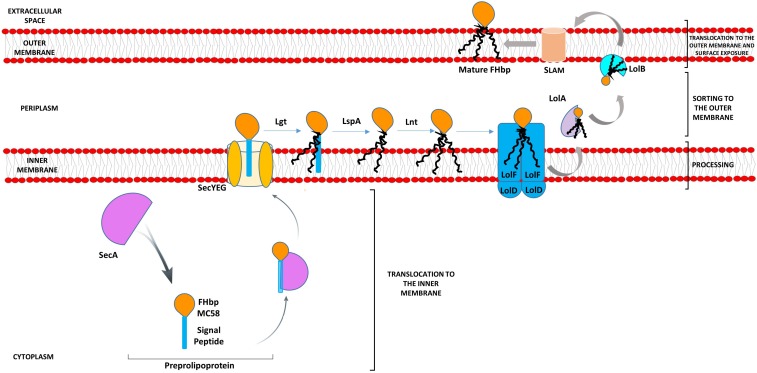
Model pathway for translocation, processing, sorting, and export of FHbp in *Neisseria meningitidis*. Preprolipoproteins are synthesized in the cytoplasm and as they emerge from ribosomes bind chaperones such as SecA, which translocate them across the IM via SecYEG. Lgt disacylates the preprolipoprotein at the conserved C residue of the lipobox of the SP, next LspA cleaves the SP exposing the C residue for further acylation by Lnt (da Silva et al., 2017). The Lol apparatus (LolFD) in *N. meningitidis* sorts the triacylated lipoprotein to the OM by delivering this to chaperone LolA, which releases the mature lipoprotein to the OM lipoprotein acceptor, LolB (da Silva et al., 2017). SLAM then localizes FHbp to the cell surface (Hooda et al., 2016).

Lipoproteins, such as FHbp, are synthesized as precursors (preprolipoproteins) in the cytoplasm, which are subsequently taken through a sequential pathway for processing and sorting to the outer membrane (OM) (Kovacs-Simon et al., 2011; da Silva et al., 2017). The N-terminal signal peptide (SP), characteristic of bacterial lipoproteins, comprises a positively charged n-region, a hydrophobic h-region and a c-region with the consensus sequence [LVI][ASTVI][GAS] followed by an invariant C residue, known as the lipobox (Babu et al., 2006). Translocation of the preprolipoprotein across the inner membrane (IM) occurs predominantly via the general secretory or Sec pathway (Driessen and Nouwen, 2008). Both the n-region and h-region are involved in interaction with SecA or other chaperones which deliver the precursor protein to the Sec-YEG transmembrane channel (Mori et al., 1997). Preprolipoprotein diacylglyceryl transferase, Lgt, transfers the diacylglyceryl group from phosphatidylglycerol to the conserved C residue (Sankaran and Wu, 1994). This diacylglyceryl modification of preprolipoproteins is vital for substrate recognition by the dedicated lipoprotein signal peptidase LspA which cleaves the SP (Tokunaga et al., 1982; Inouye et al., 1983; Vogeley et al., 2016). In diderms, such as *Neisseria*, the amino group of the C residue is further modified with a third acyl chain through apolipoprotein N-acyltransferase, Lnt (da Silva et al., 2017). Fully processed lipoproteins (cleaved and lipidated) that are destined to be anchored to the OM are transported by the Lol (lipoprotein OM localization) machinery (Tokuda, 2009). We previously showed that in *N. meningitidis*, Lol consists of the IM ABC transporter-like complex, LolFD (as opposed to LolCDE of *Escherichia coli*) the periplasmic LolA chaperone and the OM LolB lipoprotein receptor (da Silva et al., 2017) (Figure 1). FHbp is then surface localized by lipoprotein assembly modulator (Slam) (Hooda et al., 2016). Slam either acts as a conduit for FHbp or delivers it to another OM protein or to a complex such as Bam (Hooda et al., 2016) (Figure 1).

Through an accelerated approval process, both Trumemba (Pfizer) and Bexsero (GSK) were licensed by the FDA in 2014 and 2015 respectively for immunization to prevent invasive disease by meningococcal group B in the United States in individuals 10 to 25 years of age. Trumenba comprises two recombinant FHbps, one from subfamily A, the other from subfamily B, both containing the lipid moiety found in the native protein (Fletcher et al., 2004; Gandhi et al., 2016). A recombinant non-lipidated form of FHbp from subfamily B is also one of the antigens of the Bexsero vaccine (GSK) (Vernikos and Medini, 2014) licensed for infants from 2 months of age in Europe in 2013 and, like Trumenba, now licensed globally (Basta and Christensen, 2016).

A concern however for FHbp-based vaccines is the variation in expression level of FHbp between strains by over 15-fold and a threshold level of expression of at least 757 molecules of FHbp per cell is required for killing by FHbp-specific antibodies, meaning that isolates with low FHbp surface decoration fail to be effectively targeted (Jiang et al., 2010; Zlotnick et al., 2015; Biagini et al., 2016). The regulation of FHbp expression is influenced by external factors; at the transcriptional level by both iron and oxygen availability and at the translational level, by temperature (Oriente et al., 2010; Sanders et al., 2012; Loh et al., 2016). Our work has focused on bacterial-cell-intrinsic molecular factors, which govern the processing and localization of FHbp to the surface (da Silva et al., 2017) (Figure 1) which is key for target recognition following immunization with FHbp-based vaccines.

**FIGURE 2:**
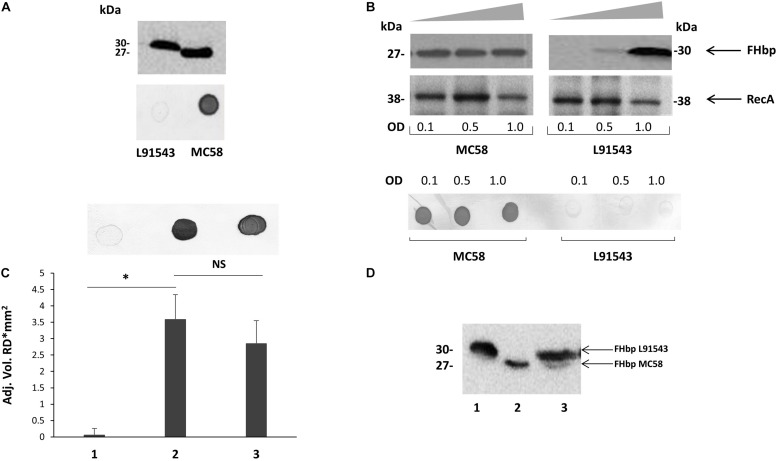
Cleavage and surface localization of FHbp in L91543 upon transformation with MC58 *fHbp*. WCL **(A,B,D)** and whole cell suspensions from fresh plate cultures **(A,C)**, or from broth cultures **(B)**, analyzed by Western immunoblot and immuno-dot blot respectively with JAR4. **(A)** Western immunoblot (upper panel) and immuno-dot blot (lower panel) of strains L91543 and MC58. **(B)** Different growth phases of broth cultures of MC58 and L91543 analyzed by Western immunoblot (upper panel), including anti-recA antibody to verify loading control, and immuno-dot blot (lower panel), representative of three independent experiments. **(C)** Immuno-dot blot and **(D)** Western immunoblot: Lanes; 1, L91543; 2, MC58; 3, L91543*fHbp*MC58. Data are representative of five independent experiments. For **(C)**, the reflective density was measured by a GS-800^TM^ calibrated densitometer. The one way ANOVA followed by Dunnett’s test was employed for statistical analysis in GraphPad (V. 6). All columns represent mean ± SEM, ^∗^*p* < 0.05 vs. strain MC58.

One of the studies correlating surface exposure of FHbp and susceptibility to killing by anti-FHbp antibodies was conducted by Newcombe et al. (2014) [27]. While strain L91543 displayed very little FHbp at the surface and demonstrated lack of killing by anti-FHbp sera, strain MC58, a well-characterized reference strain, demonstrated strong surface expression of FHbp and efficient killing by anti-FHbp sera (Newcombe et al., 2014). We further observed a difference in size in FHbp between these two strains, despite their high amino acid (AA) sequence identity across the same length open reading frame (Figure 2A) (Karlyshev et al., 2015). The FHbp of strain MC58 is of the expected size; 27 kDa (Schneider et al., 2009) and is known to be tri-palmitoylated at the C residue (Mascioni et al., 2010) whereas the FHbp of L91543 is about 3 kDa larger (Figure 2A) (Karlyshev et al., 2015). We identified two non-synonymous single nucleotide polymorphisms (SNPs) in the h-region of the FHbp SP of L91543 that we hypothesized affects translocation and processing and ultimately localization to the cell surface (Karlyshev et al., 2015). In this study, by restoring these SNPs we provide strong evidence to support our hypothesis.

Surprisingly we found that the majority of invasive isolates circulating in the United Kingdom carry these SP SNPs and exclusively express the preprolipoprotein, which is partially retained in the cytoplasm due to reduced binding to SecA. Of the precursor protein that is translocated to the IM, some is retained in the periplasm and some exported to the surface. Our data suggest translocation to the OM is facilitated by Lnt and surface display is conducted by Slam following escape from processing by Lgt and LspA. Our findings thus suggest novel roles for Lnt as a chaperone facilitating periplasmic transport and for Slam in localizing non-lipidated FHbp to the cell surface. We show that the reduced surface display of precursor FHbp, due to cytoplasmic retention, correlated with reduced killing by FHbp-specific antibodies. Knowledge of the SP sequence of isolates in addition to the promoter and upstream regulatory sequence of FHbp (Cayrou et al., 2018) will enable improved prediction of surface abundance and thus coverage by Trumenba. New algorithms can now be deployed to predict whether other preprolipoproteins are processed to become mature lipoproteins or remain as precursors. This manuscript has been released as a Pre-Print at BioRxiv (da Silva et al., 2019).

## Materials and Methods

### Bacterial Strains and Culture Conditions

*Escherichia coli* strain JM109 single use competent cells were purchased from Promega and used for transformations in *E. coli*. BTH101 (Euromedex) was used as a reporter strain for BACTH assays.

The two reference (control) strains of *N. meningitidis* used in this study are MC58, serogroup B:15:P1.7,16, ST-74; ET-5 purchased from LGC Standards and L91543 serogroup C:2aP1.2, ST-11; ET-37 kindly provided by Professor McFadden (University of Surrey) (Table 1). Strain H44/76, serogroup B:P1.7,16:F3-3: ST-32 was gifted by Rob Read and all other group B isolates were given by Christopher Bayliss with approval from Ray Borrow (Public Health England) (Table 2) and are listed in the Meningococcus Genome Library database (MRF collection). These isolates were obtained from patients in England, Wales, Northern Ireland and Scotland from 2009 to 2017.

**TABLE 1:** Frequency of different FHbp SP classes in capsular group B isolates in the MRF Meningococcus Genome Library database.

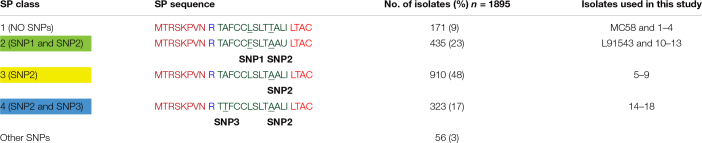

*The n-region is shown in brown, h-region in green and lipobox in red, corresponding to the display generated by the DOLOP lipoprotein prediction program (https://www.mrc-lmb.cam.ac.uk/genomes/dolop/analysis.shtml) (Babu et al., 2006).*

**TABLE 2:** MenB invasive isolates used in this study.

Class	Isolate number	Isolate, full name
1	–	MC58
	1	H44/76
	2	M10_240684
	3	M10_240701
	4	M02_241729
3	5	M10_240579
	6	M13_240525
	7	M04_241215
	8	M11_241066
	9	M13_240614
2	–	L91543
	10	M10_240750
	11	M13_240675
	12	M11_240236
	13	M12_240006
4	14	M11_241033
	15	M14_240367
	16	M02_240210
	17	M11_240077
	18	M13_240486

*The SP class is indicated for each isolate.*

*Escherichia coli* strains were grown at 37°C in Lysogeny broth (LB) with shaking at 200 rpm or on agar (Merck). All meningococcal strains were grown on GC agar plates or GC broth (Difco) containing Kellogg’s glucose and iron supplements (Kellogg et al., 1963) in a moist atmosphere containing 5% CO_2_ at 37 or 30°C for transformation experiments and with shaking at 220 rpm for broth cultures.

Antibiotics were purchased from Sigma and added at the following concentrations: kanamycin, 30 and 60 μg/ml, erythromycin, 300 and 0.3 μg/ml for *E. coli* and *N. meningitidis* respectively; 100 μg/ml ampicillin for *E. coli;* and 30 μg/ml nalidixic acid for *E. coli* BTH101.

### Antibodies

Mouse anti-FHbp-Mabs, JAR4 and JAR5, were obtained from NIBSC. JAR4 is IgG2a and JAR5 is IgG2b; both were obtained from mice immunized with recombinant FHbp derived from MC58 (Welsch et al., 2004). Mouse anti-FHbp polyclonal antibody was kindly provided by Christoph Tang and rabbit anti-RecA IgG was purchased from Abcam. Factor H protein was purchased from Bio-Rad and the mouse monoclonal IgG1 Factor H Antibody (OX24) conjugated with PE purchased from Santa Cruz Biotechnology. Secondary antibodies included donkey anti-rabbit HRP-linked IgG, sheep anti-mouse HRP-linked IgG (GE Healthcare, United Kingdom) for Western immunoblotting and rat anti-mouse IgG H + L conjugated with FITC for FACs analysis (Thermo Fisher Scientific).

### SDS-PAGE, Western Immunoblotting, and Immuno-Dot Blotting

Whole cell lysates (WC) were prepared from broth or plate cultures resuspended in PBS fractionated by 12 or 16% (w/v) SDS-PAGE and immunoblotted as described previously (da Silva et al., 2017). For immunoblotting, membranes were incubated with JAR4 (diluted 1:1000 from 1 mg/ml stocks) or rabbit anti-RecA antibody diluted (1:1000) for 1 day, washed then incubated with either sheep anti-mouse or donkey anti-rabbit HRP-linked secondary antibody. Protein bands were detected by enhanced chemiluminescence (GE Healthcare, United Kingdom) or 3,3′,5,5′-tetramethylbenzidine (TMB) (Sigma). Band intensity was quantified using a GS-800^TM^ calibrated densitometer (Bio-Rad) or ImageJ 1.x (Schneider et al., 2012) calibrated to perform Optimal Density (OD) based on a pallet of colors in grayscale. Immuno-dot blots were performed as described previously (da Silva et al., 2017).

### Palmitate Labeling of Lipidated Proteins

Bacterial cultures were grown in supplemented GC broth then after an initial doubling period, alkyne-labeled palmitic acid (Cayman Chemical) was added to a final concentration of 45 μM. Bacteria were incubated for at least four more hours at 37°C.

### Immuno-Precipitation of FHbp From Precipitated Supernatant

Samples were immuno-precipitated with Protein G Mag Sepharose (GE Healthcare Life Sciences) and Mab JAR4. Following incubation of 100 μl of precipitated sample with 5 μg of JAR4 overnight at 4°C, samples were incubated for 1 h with 100 μl of magnetic bead slurry. Using a magnetic particle concentrator (MPC), beads were washed twice with PBS and FHbp recovered following the addition of 100 μl 0.1M glycine-HCl (pH 2.5 to 3.1). Buffer exchange from glycine-HCl buffer to click reaction buffer (100 mM Na-Phosphate Buffer, pH 7) was performed with Slide-A-Lyzer Dialysis cassettes (Sigma).

### Click Chemistry

The Click Chemistry labeling system “CuAAC Biomolecule Reaction Buffer Kit (THPTA based)” (Jena Bioscience) was used following manufacturer’s instructions (Ostberg et al., 2013). FHbp was coupled to biotin azide (Stratech) then samples fractionated on 10–20% (w/v) SDS-PAGE gels (Novex). Western immunoblotting was performed by incubating the membrane with Streptavidin HRP-linked protein in PBS-BSA buffer and developing with TMB (Sigma).

### Harvesting Cellular Compartments of *N. meningitidis*

Periplasmic extracts were prepared using a method previously described (Ames et al., 1984). Cells from over-night GC plate cultures were suspended to *A*_600_ 1.0 in 500 μl buffer (50 mM Tris-HCl pH 8.0) and pelleted at 3,500 × *g* for 2 min. The pellet was resuspended in 200 μl of the same buffer and 20 μl of chloroform was added. After brief vortexing, tubes were incubated for 15 min at room temperature. After centrifugation at 6,500 × *g* for 2 min, the upper portion of the supernatant, containing the periplasmic proteins, was carefully aspirated and placed in a second tube. The pellet containing the remaining IM, OM, and cytoplasmic proteins was partitioned following approaches adapted from two different research groups (Clark et al., 1987; Rahman et al., 2000). The pellet was re-suspended in 500 μl buffer [50 mM Tris-HCl pH 8.0, 20% (w/v) sucrose] containing 1 mg/ml lysozyme and incubated for 30 min at 4°C. After two cycles of freeze-thawing, the cells were subjected to sonication (2 bursts of 30 s). Cellular debris were removed by centrifugation at 9,500 × *g* for 10 min. Ultra-centrifugation at 100,000 × *g* for 60 min enabled partitioning of the membranes in the pellet from cytoplasmic proteins in the supernatant. The IM was selectively solubilized by treatment with 200 μl of 1% (w/v) sodium lauroyl sarcosinate in 10 mM HEPES (*N*-2-hydroxyethylpiperazine *N*′-2-ethanesulfonic acid) pH 7.4 buffer. After centrifugation at 100,000 × *g* for 1 h the supernatant containing solubilized IM proteins was separated from the pellet containing OM proteins. The pellet was washed with ethanol and resuspended in 200 μl PBS.

### Molecular Methods for DNA Manipulations

Genomic DNA was extracted from *N. meningitidis* using the Gentra Puregene Yeast/Bact Kit (Qiagen) and plasmid DNA was extracted from *E. coli* using the QiaPrep Spin kit (Qiagen). DNA samples were analyzed by agarose gel electrophoresis and visualized by staining with SYBR Safe (Invitrogen). Restriction enzymes (NEB), T4 DNA ligase (Promega), and Antarctic Phosphatase (NEB) were used according to the manufacturer’s recommendations. PCRs were performed using Q5 polymerase kit (NEB) in a Perkin- MJ Research PTC-200 Peltier Thermal Cycler or C1000 Touch^TM^ Thermal Cycler (Bio-Rad). Primers were purchased from Sigma and their sequences listed in Table 3. PCR products and restriction digested DNA were purified using the PCR Mini Elute kit (Qiagen). *E. coli* was transformed by heat shock (Froger and Hall, 2007).

**TABLE 3:** PCR primer pairs used in this study.

	**PCR, sequencing and RT-PCR**
**Primer name**	**Primer sequence**
***fHbp* cloning (restriction sites underlined)**	
*Pac*I-fHbp-for	5′-GCGCAATTAATTAATTGCTTCTTTGACCTGCC-3′
*Pme*I-fHbp-rev	5′-ACCTGTTTAAACAATGGTTATTGCTTGGCG-3′
**pGCC4 sequencing primers**	
pGCC4-fwd	5′-AGACATCCGCCAAACCATCC-3′
pGCC4-rev	5′-TGCTTCCGGCTGTTGTGTGG-3′
**RT-PCR primers**	
fHbp-for	*5*′*-*GTTTCGCAACCATCTTCCCG*-3*′
fHbp-rev	*5*′*-*GACTTTATCCGTCAAATCGA*-3*′
recA-for	*5*′*-*GAAGAGGTATTGGCAACGA*-3*′
recA-rev	*5*′*- CGGA*TTTGTTGATGATGTCG*-3*′
**Bacterial two-hybrid (restriction sites underlined)**	
BamHIfwd_MC58FHbp	5′-GCGAGGATCCATGACTAGGAGTAAACCTGTGAATC-3′
EcoRIrev_MC58FHbp	5′-GATCGAATTCTTATTGCTTGGCGGCAGGCCGATATG-3′
BamHIfwd_L91543FHbp	5′-GCGAGGATCCATGCCGTCTGAACCGTTGTTCGGACGGC-3′
EcoRIrev_L91543FHbp	5′-GATCGAATTCTTACTGCTTGGCGGCAAGACCGATATGG-3′
PstIfwd_SecA	5′-CGATCTGCAGATGCTGACAAACATTGCCAAGAAAATC-3′
SmaIreverse_SecA	5′-CGACCCGGGTTAAGCCAGTTTGCCGTGGCATTG-3′
**Site-directed mutagenesis (incorporated mutations italicized)**	
SP1_Fwd	5′-CTTCTGCTGC*CTT*TCTCTGACCG-3′
SP1_Rev	5′-GCAGTTCGGTTCACAGGT-3′
SP2_Fwd	5′-TTCTCTGACC*ACT*GCCCTGATTC-3′
SP2_Rev	5′-AAGCAGCAGAAGGCAGTT-3′
**Deletion of *fHbp* by SOEing**	
HA1_FHbp_Fwd	5′-GATAGAATTCCGAGTATGCAGCTTTG-3′
Rev_HA1_FHbp	5′-***GATGATGGTTG****CCATTGTGAA*AATGCCGTCC-3′
Kan_fwd	5′-*TTCACAATGG****CAACCATCATC*GATG**-3′
Kan_rev	5′-*AAACCT****TTCAGACGGCAT*GTAATGCTCTGCC**-3′
HA2_FHbp_fwd	5′-***ATGCCGTCTGAA****AGGTTT*ACTCCTAGTCATACG-3′
HA2_FHbp_rev	5′-CTTAGGATCCCCACGGCGCATACAAATTC-3′
**Deletion of *slam* by SOEing (*kan* sequence in bold, HA sequence underlined, overlapping sequence italicized)**	
HA1_0313_Fwd	5′-GATAGAATTCAGGCGCAGTTTACCTACTTG-3′
HA1_0313_rev	5′-***GATGATGGTT****GTATCAATC*GGCGGATTGTATC-3′
0313_Kan_fwd	5′-*GATACAATCCGCCGATTGATA****CAACCATCAT*CGATG**-3′
0313_Kan_rev	5′-*AACAGCAA****TTCAGACGGCAT*GTAATGCTCTGCC**-3′
HA2_0313_fwd	5′-***ATGCCGTCTGAA****TTGCTGTT*CCTTTTCGGAGG-3′
HA2_0313_rev	5′-CTTAGGATCCGAACGGCTTATGGCTTTGGGAC-3′
**Disruption of Lnt**	
Lnt_fwd	5′-GGCAGGAGATATGCGCTAAG-3′
Lnt_rev	5′-GTACTGGTCGCCCACAACCT-3′

### Genome Sequencing

The *N. meningitidis* L91543 genome sequence was completed using a hybrid approach employing a combination of short and long reads assemblies produced by IonTorrent (Karlyshev et al., 2015) and PacBio technologies. PacBio sequencing was conducted at TGAC (The Genome Analysis Centre, Norwich, United Kingdom) using an RSII sequencing machine with P6/C4 sequencing chemistry and a single SMRT cell. When analyzing the PacBio assembly, long redundant regions were detected at the ends of the sequence. CLC Genomics Workbench software and high quality reads generated by IonTorrent PGM were used for elimination of artifactual redundancies and for circularization, as well as for final verification of the sequence and correction of any errors produced by PacBio.

### BLAST Analysis

Whole genome sequence (WGS) data from capsular group B isolates with complete FHbp profiles in the Meningitis Research Foundation Meningococcus Genome Library database (containing WGS data from all United Kingdom disease isolates from 2010 to 2017) was interrogated using the BLAST tool implemented within the PubMLST.org/neisseria database using default settings (last analyzed September, 2018).

### Construction of Strain L91543*fHbp*MC58

The region incorporating the promoter and open reading frame of *fHbp* was PCR-amplified from MC58 genomic DNA with primers *Pac*I-*fHbp*-for and *Pme*I*-fHbp*-rev (Table 3) digested with *Pac*I and *Pme*I sites and cloned into the *Pac*I, *Pme*I sites of *Neisseria* complementation vector, pGCC4 (Addgene) (Mehr and Seifert, 1998). The resulting plasmid, pGCC4*fHbp*MC58 was verified by DNA sequencing with pGCC4 primers (Table 3) and the plasmid used to transform *N. meningitidis* strain L91543 as previously described (Zhang et al., 2010) with selection on erythromycin. A transformant was verified by PCR with subsequent DNA sequencing and designated L91543*fHbp*MC58. The same approach was used to create plasmid pGCC4*fHbp*L91543 with the L91543 version of *fHbp*, which was used for site-directed mutagenesis.

### Construction of Strain L91543Δ*fHbp*

Gene Splicing by Overlap Extension (gene SOEing) was used to create a fusion PCR product to replace *fHbp* in strain L91543 with the kanamycin resistance gene (*kan*) from EZ:Tn5 < KAN-2 > insertion kit (Epicentre) following the approach described by Horton (Horton, 1995). In the first round of PCRs, homology arms (HA1 and HA2) of approximately 600 bp flanking *fHbp* were amplified from genomic DNA of L91543 using primers, HA1_FHbp_Fwd and Rev_HA1_FHbp and HA2_FHbp_fwd and HA2_FHbp_rev, and the *kan* gene was amplified using primers Kan_fwd and Kan_Rev. As shown in Table 3, in bold are the regions that bind to *kan*, underlined are the regions that bind to the HA of interest and in italics are the regions that over-lap. The HA1 and *kan* PCR products obtained were gene-cleaned then used as template for the second round of PCR with primers HA1_FHbp_Fwd and Kan_Rev and the annealed product generated cleaned and used as template along with the HA2 PCR product for a third round of PCR with primers HA1_FHbp_Fwd and HA2_FHbp_rev. The final PCR product generated containing HA1-*kan*-HA2 was gene cleaned and sequenced for confirmation. The verified construct was used to transform strain L91543 with selection on kanamycin. Deletion mutants were confirmed by PCR and DNA sequencing and designated L91543Δ*fHbp*.

### Disruption of Slam

In the same manner as that described above, the gene encoding Slam (NMB0313) was replaced with *kan*. For the first round of PCRs, primers HA1_0313_Fwd and HA1_0313_rev and HA2_0313_fwd and HA2_0313_rev were used to amplify approximately 600 bp of sequence flanking *slam* from genomic DNA of MC58 and *kan* was amplified with primers 0313_Kan_fwd and 0313_Kan_rev (Table 3). The appropriate primer pairs were used in second and third round PCR and the construct confirmed by sequencing then used to transform MC58, L91543 and isolates 6 and 18 with selection on kanamycin. Insertion mutants were confirmed by PCR and DNA sequencing and designated MC58Slam, L91543Slam, Isolate6Slam, and Isolate18Slam.

### Disruption of Lnt

Primers, Lnt_fwd and Lnt_rev (Table 3), annealing approximately 600 bp upstream and downstream respectively of *lnt* in strain MC58Lnt, were used to amplify *lnt* that had been disrupted by the insertion of Tn5 < KAN-2 > (Epicentre) (da Silva et al., 2017). The PCR product generated was used to transform L91543 and isolates 6 and 8 with selection on kanamycin. Deletion mutants were confirmed by PCR and DNA sequencing and designated L91543Lnt, Isolate6Lnt, and Isolate18Lnt.

### Site-Directed Mutagenesis

Site-directed mutagenesis of the *fHbp* SP in pGCC4*fHbp*L91543 to repair SNP1 and SNP2 individually was performed using the Q5^®^ Site-Directed Mutagenesis Kit (NEB) according to the manufacturer’s recommendations. SNP1 was corrected to create plasmid pGCC4L*fHbp*SNP1 and SNP2 was corrected to create plasmid pGCC4L*fHbp*SNP2. Seven ng of pGCC4*fHbp*L91543 and 0.5 μM of each pair of primers (Sigma) were used for amplification and incorporation of the desired mutation. Primers SP1_Fwd and SP1_Rev, and SP2_Fwd and SP2 _Rev (Table 3) were used to incorporate SNP1 and SNP2 respectively. PCR was performed in a C1000 Touch^TM^ Thermal Cycler (Bio-Rad) with the following thermo-cycling conditions; 98°C for 30 s followed by 25 cycles of 98°C for 10 s (denaturation), 68°C for 15 s (annealing), 72°C for 3 min and 25 s (extension) and 72°C for 2 min (final extension). One μl of the PCR product was kinase-, ligase-, and *Dpn*I- treated (KLD treatment) and incubated for 5 min at room temperature. Five μl of the KLD mix was then used to transform competent cells. After plating on LB agar with kanamycin and incubating overnight, several colonies were isolated, grown individually in LB broth with kanamycin and plasmid DNA extracted and sequenced with pGCC4 primers (Table 3) for verification. To repair both SNPs, the *Pac*I-*Pme*I fragment of pGCC4*fHbp*L91543 was commercially synthesized (Life Technologies) with the two SNPs repaired to resemble the *fHbp* SP of MC58 and the DNA cloned into the *Pac*I-*Pme*I sites of pGCC4 to create pGCC4L*fHbp*SNP1 + 2. The construct was verified by sequencing with pGCC4 primers (Table 3).

Strain L91543Δ*fHbp* was transformed with pGCC4 *fHbp*L91543 to create L91543Δ*fHbp*L*fHbp* with no SNP corrections as a negative control. The same strain was transformed with pGCC4L*fHbp*SNP1 to generate recombinant strain L91543Δ*fHbp* + L*fHbp*SNP1, with pGCC4L*fHbp*SNP2 to generate strain L91543Δ*fHbp* + L*fHbp*SNP2 and finally with pGCC4L*fHbp*SNP1 + 2 to create strain L91543Δ*fHbp* + L*fHbp* SNP1 + 2.

### Bacterial Two-Hybrid Assay (BACTH)

The protein–protein interaction of MC58 FHbp and L91543 FHbp with SecA was investigated using the Bacterial Adenylate Cyclase Two-Hybrid System Kit (Euromedex) according to manufacturer’s instructions. First, *fHbp* from MC58 and from L91543 was PCR-amplified with the primer pair BamHIfwd_MC58FHbp and EcoRIrev_MC58FHbp and primer pair BamHIfwd_L91543FHbp and EcoRIrev_L91543FHbp respectively (Table 3) then the PCR products cloned separately into vector pUT18. The gene encoding SecA from MC58 was PCR-amplified with primer pair, PstIfwd_SecA and SmaIreverse_SecA (Table 3) then cloned into vector pKT25. 25–50 ng of the appropriate prey (pKT25-based construct) and the equivalent concentration of appropriate bait (pUT18-based construct) were co-transformed into 100 μl of competent *E. coli* BTH101 cells and plated on MacConkey agar containing 0.5 mM IPTG and appropriate antibiotics. Bacteria expressing interacting hybrid proteins formed pink/purple colonies while cells expressing non-interacting proteins remained white/light pink. As a positive control, a co-transformant containing commercial pKT25-zip and pUT18-zip constructs was used. Co-transformants containing empty vector pKT25 and/or pUT18 were used as negative controls. Pink colonies were isolated and grown in LB broth and plasmid DNA extracted for verification by PCR and sequencing.

### β-Galactosidase Assay

Following the approach previously described (Chambonnier et al., 2016), to measure the level of protein–protein interaction between FHbp from MC58 or L91543 with SecA, LB broth cultures were grown to *A*_600_ 0.6 then induced for 3 h with 0.5 mM IPTG. Five μl of induced culture were mixed with 900 μl of Z buffer (0.06M Na_2_HPO_4_ 2H_2_O, 0.04M NaH_2_PO_4_, 0.01M KCl, 2 mM MgSO_4_.7H_2_O, 14.20M β-mercaptoethanol (pH 7), before addition of 20 μl of 0.1% (w/v) SDS and 100 μl of CHCl_3_ to permeabilize the cells. Substrate solution was prepared by solubilizing orthonitrophenyl-β-galactosidase (Sigma) in Z buffer without the β-mercaptoethanol to a final concentration of 4 mg/ml. 40 μl of substrate solution were added to 180 μl of Z buffer and 20 μl of permeabilized cells in a 96-well plate. The plate was incubated at room temperature for at least 20 min. Readings were taken on a microplate reader at *A*_405_ and *A*_540_ and β-galactosidase activity calculated using the equation below and expressed in Miller units.

β-galactosidase⁢a⁢c⁢t⁢i⁢v⁢i⁢t⁢y=

1000×(A-405A)540Time(min)×vol.ofcellsinassay(ml)×A600

### FACS Analysis of Isolates to Measure the Number of JAR4 Antibodies Bound per Cell (ABC) and Quantify Surface Localization of FHbp

FHbp surface expression was assessed using a MoFlo Astrios EQ, Cell Sorter (Beckman Coulter). The approach used was similar to that described previously (Biagini et al., 2016). Approximately 1 × 10^5^ bacteria were suspended in PBS containing 1% (w/v) bovine serum albumin (PBS-BSA) and incubated with JAR4 in a final volume of 100 μl for 1 h at 37°C. After two washes with PBS, JAR4 binding was detected using FITC-conjugated rat anti-mouse IgG (H + L) (Thermo Fisher) at a 1:50 dilution for an incubation period of 1 h. After the final two washes with PBS, samples were resuspended in 500 μl of PBS-BSA with 4% (w/v) formalin. The negative control consisted of cells incubated with the secondary antibody alone. Quantum^TM^ Simply Cellular^®^ anti-rat (Bang Laboratories) microspheres were used to determine the number of ABC according to the manufacturer’s instructions. Median channel values for each population of microspheres were acquired for entry into the QuickCal^®^ spreadsheet to generate a curve that enabled the acquisition of ABC for each isolate.

### FACS Analysis of Isolates With Anti-fH Antibody to Quantify fH Binding

Approximately 1 × 10^5^ bacterial cells were suspended in PBS containing 1% (w/v) PBS-BSA and incubated with fH (Bio-Rad) at a final concentration of 5 μg/mL for 1 h at room temperature. After two washes with PBS, anti-fH antibody (OX-24) conjugated to PE was added at a 1:50 dilution for 1 h. After two more washes in PBS, samples were resuspended in 200 μl of PBS-BSA with 4% (w/v) formalin. The negative control consisted of cells incubated with anti-fH antibody alone. The relative Mean of Fluorescence Intensity (rMFI) for total fluorescent cells was acquired for each sample and these values normalized against rMFI of cells incubated with secondary antibody alone.

### Susceptibility of Isolates to Killing in Serum Bactericidal Antibody Assays

The susceptibility of the isolates to complement-mediated killing with antibodies JAR4 and JAR5 was assessed using approaches adapted from previous studies (Beernink et al., 2008, 2011; Vu et al., 2012). JAR4 and JAR5 were used in combination, in place of serum antibodies, to generate bactericidal activity (Beernink et al., 2008; Welsch et al., 2008; Malito et al., 2016). Eight μg of each antibody were used to ensure maximum killing (Beernink et al., 2008). Complement was obtained from lyophilized human sera (Sigma) reconstituted in sterile water as per manufacturer’s instructions. Complement and antibodies were added to IgG-depleted human sera (Stratech) that had been heat-inactivated for 30 min at 56°C. The bactericidal activity, i.e., the percentage of killing by the antibodies for each isolate was then determined from the CFU counts after 60 min incubation in the reaction mixture and compared with CFU counts from negative control wells at time 0.

### Reverse Transcription PCR (RT-PCR)

Following a similar approach to that we described previously (da Silva et al., 2017), RNA was extracted from 1 ml cell suspensions of each isolate standardized to *A*_600_ 0.65 (containing approximately 2 × 10^8^ cells) using the RNeasy Mini kit (Qiagen) with enzymatic lysis and Proteinase K digestion. On-column DNA digestion was performed using the RNase Free DNase kit (Qiagen). RNA quality was assessed for genomic contamination and integrity using a NanoDrop Lite (Thermo Fisher Scientific) and running 1 μl of sample on a 1% agarose gel.

One μg of cDNA was synthesized using the QuantiTect reverse transcription kit (Qiagen) with the initial genomic wipe-out step included. RT-PCR was performed in a 50 μl reaction mixture with One Taq 2x master mix (NEB), *2*0 ng of cDNA and 0.2 μM of each primer (Sigma). For amplification of cDNA of *fHbp*, *fHbp*-for, and *fHbp*-rev primers were used and for amplification of *recA*, *recA*-for, and *recA*-rev primers were used (Table 3). To qualitatively check for different levels of expression, PCR was performed in a C1000 Touch^TM^ Thermal Cycler (Bio-Rad) with the following thermo-cycling conditions; 94°C for 30 s followed by 35 cycles of 94°C for 15 s (denaturation), 56°C for 15 s (annealing) and 68°C for 20 s (extension). PCR products were visualized on a 1% agarose gel.

### Statistical Analysis

Data are shown as mean ± SEM. Multiple comparisons between groups were performed by one way ANOVA followed by Dunnett’s test. Unpaired *t*-tests with Welsh’s corrections were used for comparisons between two groups. A value of *p* ≤ 0.05 was considered statistically significant. *Post hoc* tests were run if F achieved *p* < 0.05 and there was no significant variance. All statistical analyses were performed in GraphPad Prism version 6.00 for Windows. No statistical methods were used to predetermine sample size. The experiments were not randomized other than for selection of clinical isolates used in this study. The investigators were not blinded to allocation during experiments and outcome assessment.

## Results

### SP SNPs in FHbp Prevent Cleavage but Not Surface Localization in Strain L91543

*Neisseria meningitidis* strain MC58 was originally isolated from a patient in the United Kingdom with meningococcal meningitis (McGuinness et al., 1991) and is a fully-sequenced, commonly used laboratory reference strain (Tettelin et al., 2000). Strain L91543 was isolated from the cerebrospinal fluid of a 14-year old with meningococcal meningitis in the United Kingdom (Oliver et al., 2002). The FHbp-specific monoclonal antibody, JAR4, used throughout this study was sourced from the National Institute for Biological Standards and Controls (NIBSC). JAR4 binds to an N-terminal epitope including the AA residues DHK at positions 25 to 27 (Beernink and Granoff, 2009) (Supplementary Table S1) and was generated following immunization of mice with MC58 FHbp (Welsch et al., 2004). The FHbps of both MC58 and L91543 are classified as subfamily B (Fletcher et al., 2004; Beernink and Granoff, 2009) (variant 1) (Masignani et al., 2003) and, as we previously reported, they share nucleotide and AA identities of 95 and 93% respectively (Karlyshev et al., 2015).

We investigated differences in the surface localization of FHbp between these strains at different growth phases in a whole cell immuno-dot blot assay. A consistent strong level of surface expression of FHbp was shown for MC58 during growth in contrast to L91543, which demonstrated a uniform low level of surface localization (Figures 2A,B). Western immunoblot analysis of whole cell lysates showed a consistent high level of total FHbp expression for MC58, whereas the presence of FHbp was minimal in mid-log phase and strong only at stationary phase for L91543 cultures (Figures 2A,B). Thus for L91543, the demonstration of strong total FHbp level at stationary phase yet poor surface expression implies that FHbp is retained within the cells of this strain and fails to either sort to the OM and/or become surface-exposed, unlike MC58.

We previously reported that the FHbp of L91543 appears to be 3 kDa larger than that of MC58 upon fractionation of whole cell lysates as shown in Figure 2A, this additional mass corresponding to the size of the 26 AA SP (Karlyshev et al., 2015). To investigate any defects in LspA, which should cleave this SP, or aberrations in other proteins involved in the sequential pathway for FHbp processing and export (da Silva et al., 2017) (Figure 1), the genome of L91543 was fully sequenced (GenBank accession number CPO16684) and BLAST analysis performed using relevant query sequences from MC58. LspA is 100% identical between the two strains and all other proteins share > 97% identity with no evidence of frameshifts or premature stop codons, whereas FHbp exhibits only 93% AA identity between these two strains (Supplementary Table S2). Notably the SP region of FHbp exhibits even greater divergence due to the presence of 2 SNPs in L91543; leucine (L) substituted by phenylalanine (F) at position 15 (SNP1) and threonine (T) substituted by alanine (A) at position 19 (SNP2) (Table 1).

To test our hypothesis and determine whether differences in the FHbp sequence are solely responsible for the failure to cleave and export FHbp in strain L91543, the *fHbp* gene including its upstream promoter and ribosomal binding site from MC58 was inserted into the chromosome of strain L91543. This region was first cloned into the *Neisseria* complementation vector, pGCC4, which allows integration of DNA in the intergenic region between *aspC* and *lctP* without causing polar affects (Mehr and Seifert, 1998; da Silva et al., 2017). Plasmid pGCC4*fHbp*MC58 was transformed into strain L91543 and JAR4 was used to assess FHbp expression. Immuno-dot blot of whole cells showed that the recombinant strain, L91543*fHbp*MC58, acquired the ability to express FHbp at the cell surface (Figure 2C). Western immunoblot analysis of L91543*fHbp*MC58 revealed two bands: a lower band with a mobility equivalent to the cleaved FHbp of MC58 and a higher molecular weight band corresponding to the expected mobility for the native, uncleaved protein (Figure 2D).

**FIGURE 3:**
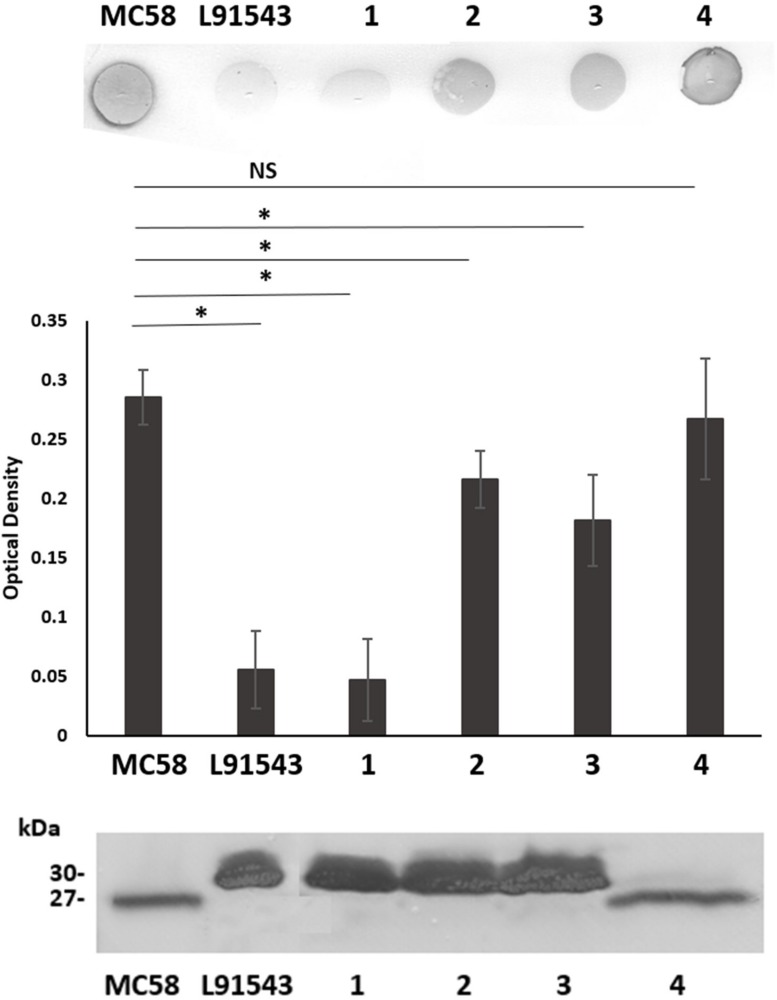
SP SNPs in FHbp prevent cleavage but not surface localization. Whole cell suspensions and WCL from fresh plate cultures analyzed by immuno-dot blot (upper panel) and Western immunoblot (lower panel) respectively with JAR4. Lanes; 1, L91543Δ*fHbp* + L*fHbp*; 2, L91543Δ*fHbp* + L*fHbp*SNP1; 3, L91543Δ*fHbp* + L*fHbp*SNP2; 4, L91543Δ*fHbp* + L*fHbp*SNP1 + 2, representative of three independent experiments. The density of spots was measured by ImageJ and the one way ANOVA followed by Dunnett’s test was employed for statistical analysis in GraphPad (V. 6). All columns represent mean ± SEM, ^∗^*p* < 0.05 vs. strain MC58.

To investigate the specific and combined contribution of the two individual SNPs in L91543 SP on cleavage and export, first *fHbp* was deleted in L91543 (L91543Δ*fHbp*) then complemented with wild type (L91543Δ*fHbp* + L*fHbp*) or versions with site-directed alteration in the SP (L91543Δ*fHbp* + L*fHbp*SNP1, L91543Δ*fHbp* + L*fHbp*SNP2, and L91543Δ*fHbp* + L*fHbp*SNP1 + 2). Western immunoblot analysis confirmed complete loss of FHbp expression in L91543Δ*fHbp* (Supplementary Figure S1). L91543Δ*fHbp* + L*fHbp*SNP1 and L91543Δ*fHbp* + L*fHbp*SNP2 showed moderate levels of surface expression and L91543Δ*fHbp* + L*fHbp*SNP1 + 2 showed a level of surface display comparable to MC58 (Figure 3). Interestingly Western immunoblot analysis showed faster mobility, indicative of SP cleavage, for only strain L91543Δ*fHbp* + L*fHbp*SNP1 + 2, which had both SNPs corrected but not for either recombinant strain with individual SNPs corrected (Figure 3). Our results suggest that both AA substitutions impact on the FHbp translocation-processing pathway at some point up to, or at, the SP cleavage step and surprisingly that repair of either SNP individually can permit partial localization of FHbp to the cell surface without the need for prior SP cleavage. This was not expected for this sequential canonical lipoprotein processing pathway.

### FHbp With SP SNPs Shows Reduced Binding to SecA

**FIGURE 4:**
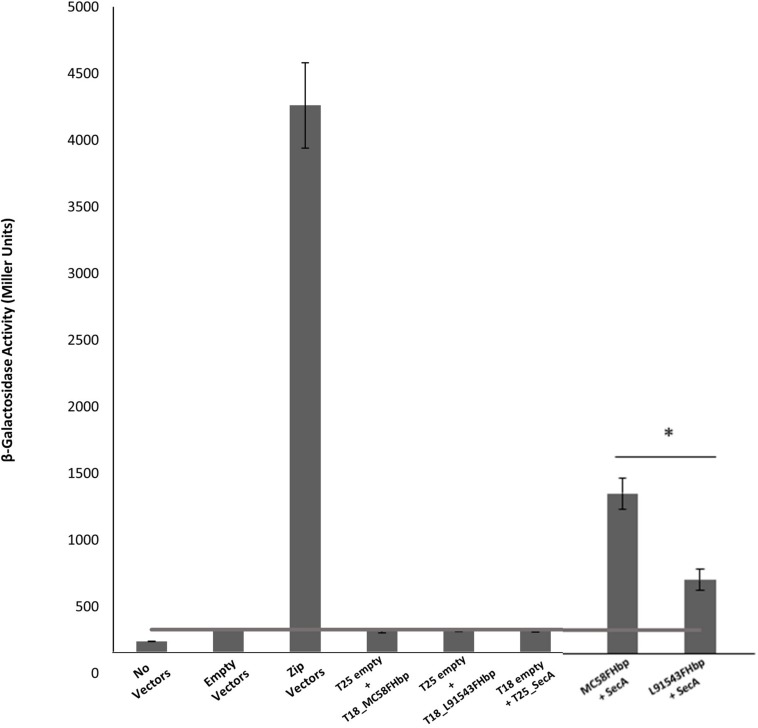
FHbp with SP SNPs shows reduced binding to SecA. Bacterial two hybrid experiments to compare FHbp binding to SecA in MC58 and L91543. The *fHbp* gene of strains MC58 and L91543 and *secA* gene of MC58 were cloned into the two-hybrid pUT18 (T18-prey) and pKT25 (T25-bait) vectors respectively and the appropriate prey and bait pair of vectors co-transformed in BTH101. In parallel, zip prey and zip bait vectors were co-transformed in BTH101 as a positive control for protein–protein interaction. Likewise, different prey-bait combinations but with one vector or both vectors empty were used as negative controls. The interactions were quantified by measuring the β-galactosidase level expressed in Miller units (values taken from four independent clones). Unpaired *t-*tests with Welsch’s correction were conducted to compare the interactions. All columns represent mean ± SEM, ^∗^*p* < 0.05 vs. MC58 FHbp.

Most proteins destined for the OM are first targeted to the IM translocase, Sec-YEG, by the chaperone ATPase motor, SecA. SecA recognizes SPs by having a high affinity for their hydrophobic binding grooves and by electrostatically trapping their positively charged n-region through acidic residues (Gelis et al., 2007; Gouridis et al., 2009). We investigated the binding of L91543 FHbp (carrying SNP1 and SNP2) to meningococcal SecA and compared this to MC58 FHbp, which is known to undergo cleavage and processing (Masignani et al., 2003; da Silva et al., 2017). The bacterial two-hybrid approach was taken which relies on induced expression of the two heterologous proteins using *E. coli* as the host organism. The cytoplasmic readout for binding affinity in this heterologous expression system is expected to be similar to that in the meningococcus, prior to translocation to the IM. Our results showed a significant 1.9-fold lower binding of SecA to L91543 FHbp as compared to MC58 FHbp (Figure 4).

### Clinical Isolates With FHbp SP SNPs Show SP Retention Yet Surface Localization

We next investigated the prevalence of FHbp containing SP SNPs (that differ from the MC58 sequence) in the genome sequences of all 1,895 invasive group B United Kingdom isolates in the Meningitis Research Foundation (MRF) Meningococcus Genome Library database collected between 2009 and 2017. Unexpectedly we found that only 9% of isolates have a SP identical to that of MC58 (referred to as class 1) with the remaining isolates carrying either (i) both SNP1 and SNP2, like L91543 (class 2, 23%); (ii) SNP2 alone (class 3, 48%); (iii) SNP2 and a third SNP (SNP3) (class 4, 17%), or (iv) other SNPs (3%) (Table 1). All SNPs are located exclusively in the h-region 13 AA in length, and common to classes 2, 3, and 4 is SNP2 (i.e., T substituted by A at position 19).

To test if our findings for recombinant strains of L91543 apply to these MRF isolates with non-class 1 FHbp, a selection of isolates from each class were randomly selected (Table 2). Strain H44/76 was chosen as an additional positive control strain since its FHbp including SP is identical to that of MC58. FHbps of all 20 isolates belong to subfamily B/variant 1 (the most prevalent group) and possess the JAR4 epitope (Beernink and Granoff, 2009) (Table 2 and Supplementary Table S1).

**FIGURE 5:**
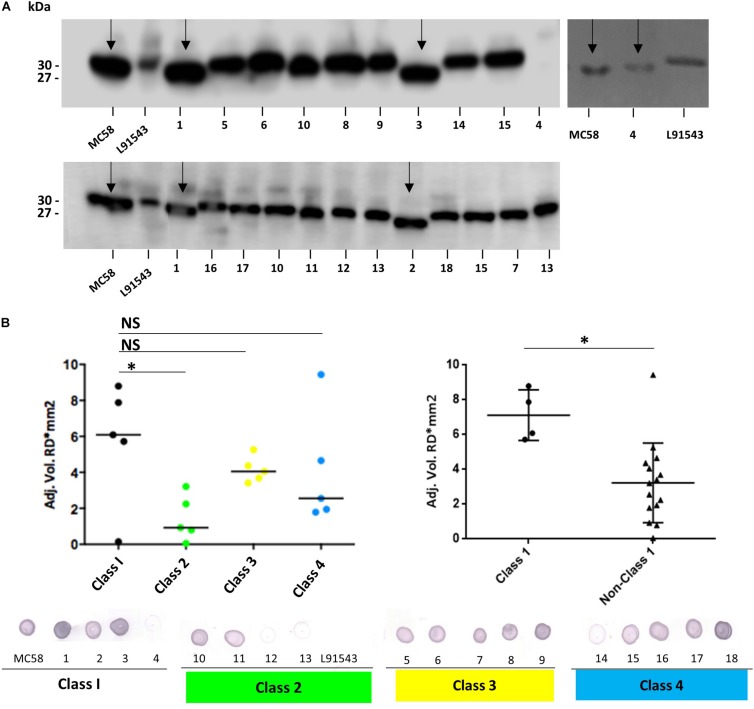
Comparison of FHbp size and surface localization in clinical isolates. For all non-class 1 isolates, the SP is uncleaved yet for most of these isolates, FHbp is surface localized. WCL or whole cell suspensions were prepared from fresh plate cultures and JAR4 used except where indicated**. (A)** Western immunoblot. For isolate 4, polyclonal anti-FHbp antibody was also used. Arrows indicate faster mobility FHbp. **(B)** Whole cell immuno-dot blot. The density of spots was measured by ImageJ and the one way ANOVA followed by Dunnett’s test employed for statistical analysis in GraphPad (V. 6). All columns represent mean ± SEM, ^∗^*p* < 0.05 vs. class 1. Pooled data of class 1 and non-class 1 were analyzed using un-paired *t*-tests with Welsch’s correction. Both columns represent mean ± SEM, ^∗^*p* < 0.05 vs. class 1.

FHbp from all isolates with class 1 SP demonstrated a similar size by SDS-PAGE to that of MC58 whilst all non-class 1 FHbp proteins had the slower mobility observed for L91543 (Figure 5A). To investigate the correlation between electrophoretic mobility and surface localization, whole cell immuno-dot blots were performed with JAR4 except for isolate 4 which JAR4 failed to recognize. For this isolate, polyclonal anti-FHbp sera was used. The median level of surface expression was highest for class 1 isolates and decreased progressively from class 3 isolates (SNP2 only), to class 4 isolates (SNP2 and SNP3) to class 2 isolates (SNP1 and SNP2) (Figure 5B). Overall, non-class 1 isolates demonstrated a significant 2.2-fold lower surface expression of FHbp than class 1 isolates (excluding isolate 4) (Figure 5B). These results indicate a failure to cleave the SP decreases the efficiency, but does not abrogate, localization of FHbp to the surface. This data support our findings for recombinant strains of L91543 (Figure 3). In contrast to the general pattern, three isolates (L91543, 12 and 13) demonstrated the presence of FHbp by Western immunoblot but no localization to the cell surface by immuno-dot blot (Figure 5B). The cause of this is explored later in this manuscript.

Interestingly all strains shown to express uncleaved FHbp in this study carry SNP2: substitution of the polar T residue with a hydrophobic A residue at position 19. The importance of polar residues in the h-region of the SP of bacterial lipoproteins first came to light in the late 1970s. In all but one of the 26 bacterial SPs known at that time, polar residues; serine (S) and/or T were found in the h-region and within close proximity to the signal peptidase cleavage site (Vlasuk et al., 1984). A T16A mutant within the SP of *E. coli* Lpp lipoprotein affected the rate of processing with accumulation of unmodified precursor in the membrane fraction (Vlasuk et al., 1984). It was postulated that this T residue either directly affected recognition by Lgt or LspA or affected IM translocation hindering accessibility of the SP by these enzymes. We questioned whether the meningococcal Lgt and LspA enzymes fail to recognize FHbp with class 2 (SNP1 and SNP2), class 3 (SNP2), and class 4 (SNP2 and SNP3) SPs (Table 1) and how these preprolipoproteins localize to the surface.

### FHbp With SP SNPs Localize to the Surface via Lnt and Slam With Escape From Processing by Lgt and LspA

**FIGURE 6:**
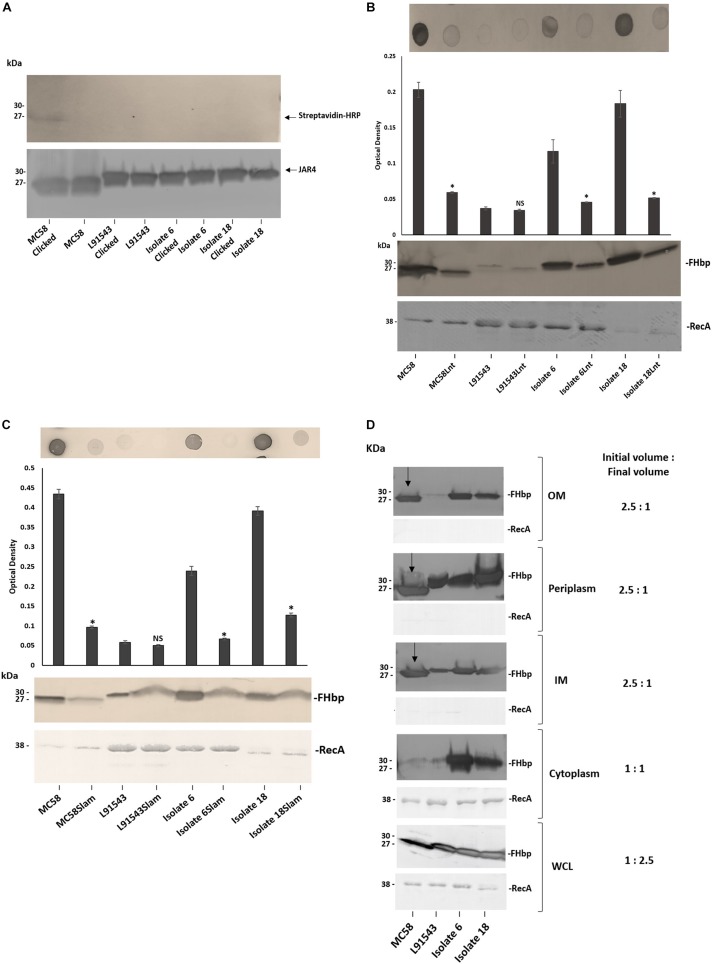
FHbp with SP SNPs localize to the surface via Lnt and Slam with escape from processing by Lgt and LspA. **(A)** Western immunoblot after growing strains in palmitic acid alkyne and clicking immuno-precipitated FHbp with biotin-azide; Streptavidin-HRP (upper panel) and JAR4 (lower panel). **(B,C)** Immuno-dot blot of broth cultures (*A*_600_ 0.5) and Western immunoblot of WCL with JAR4. For immuno-dot blot densitometer values, unpaired *t*-tests with Welsch’s correction were conducted to compare the isogenic strains. All columns represent mean ± SEM, ^∗^*p* < 0.05 vs. wild type isogenic strain. **(D)** Western immunoblot of different cellular fractions with JAR4. (Lane 1, MC58; 2, L91543; 3, isolate 6; 4, isolate 18.) **(B–D)** Equal protein loading was confirmed by the determination of RecA protein in each sample.

To test if strains with SP SNPs in FHbp are affected in lipid modification, one isolate representative of each SP class was tested for the presence of the lipid moiety. These included MC58 (class 1), L91543 (class 2), isolate 6 (class 3) and isolate 18 (class 4) (Tables 1, 2). The strains were grown in medium containing palmitic acid alkyne, FHbp was immuno-precipitated then conjugated (clicked) with biotin azide, which attaches exclusively to proteins carrying this alkyne. Western immunoblot analyses were performed separately with streptavidin and with JAR4. A band of the expected mobility for cleaved FHbp was detected by streptavidin for MC58 clicked with biotin azide and not for the corresponding non-clicked control as expected (Figure 6A). However, no bands were detected for L91543, isolate 6 or 18 whether clicked or non-clicked, suggesting that the FHbp molecules of these three isolates are not lipidated. The absence of lipid by Lgt activity would explain the subsequent failure of LspA to cleave the SP of these isolates (Figure 5A). A recent study in *E. coli* showed that prevention of diacylglyceryl modification at the conserved cysteine residue of Lpp, by Lgt depletion, prevented Lsp from processing the SP as inferred from its electrophoretic mobility (May et al., 2019). This supports earlier studies that demonstrated that substitution of the Cys residue of Lpp prevented diacylglyceryl modification with accumulation of Lpp exclusively of slower mobility and of the size expected for the unprocessed preprolipoprotein (Inouye et al., 1983).

Following failure of Lgt and LspA to lipidate and process FHbp in these non-class 1 strains, we expected the remaining canonical pathway not to be further employed and for the preprolipoprotein to be translocated to the OM and surface exposed by other mechanisms. To verify discontinuation of the canonical pathway, the *lnt* gene of the four strains was disrupted. Other than for the L91543 isogenic strains which both showed the expected low level of FHbp, the Lnt mutant version of the other three strains showed a significant reduction in surface localization as well as diminished total FHbp (Figure 6B). This supports our previous study where we showed that MC58Lnt employs mechanisms to reduce FHbp levels thereby preventing the toxic accumulation of the diacylated lipoprotein in the periplasm (da Silva et al., 2017). Our data thus suggests that in addition to the established role of Lnt in triacylating the N-terminal Cys residue in class 1 strains with intact SP (as shown for MC58) (da Silva et al., 2017), Lnt plays a role in delivering FHbp to the OM without the requirement for prior processing by LspA and Lgt.

Given that the canonical pathway appears to resume by Lnt, we questioned whether Lol was employed to translocate FHbp of these strains to the OM however it was not possible to mutate any of the Lol-encoding genes. We next investigated if Slam plays a role in the surface localization of FHbp in these strains. Again with the exception of the poor FHbp expressor, L91543, the disruption of Slam resulted in significantly reduced surface localization of FHbp as well as some reduction in total FHbp (Figure 6C). This suggests that Slam plays a role in surface localization of FHbp regardless of whether FHbp is processed or not. This contradicts what was proposed by Hooda et al. (2016) that Slams are responsible for transport of surface lipoproteins that possess a lipid anchor that needs to be flipped from the inner leaflet to the outer leaflet of the OM (Hooda et al., 2016). Rather, in this study we show that surface localization by Slam is not restricted to lipoproteins.

Given our finding that FHbp with SP SNPs binds less well to SecA, affecting its translocation efficiency to the IM, yet can be translocated to the OM and subsequently surface localized by Slam, we would expect this to be reflected in the sub-cellular distribution of FHbp. In MC58 a processed FHbp protein of 27 kDa was found in the IM, periplasm and OM but was barely detectable in the cytoplasm, providing strong evidence of highly efficient translocation from cytoplasm to the IM but less efficient translocation to the OM (Figure 6D). In contrast, for isolates 6 and 18, unprocessed precursor was detected in all four cellular compartments; cytoplasm, IM, periplasm and OM, and at high levels in all (Figure 6D). These findings demonstrate that the efficiency of translocation from cytoplasm to IM is reduced in these isolates supporting our BACTH data (Figure 4) but their OM translocation efficiency is comparable with that of MC58. For strain L91543, the total, unprocessed precursor FHbp was noticeably less abundant and all of this localized to the IM and periplasm suggesting complete translocation across the IM (Figure 6D). Conversely, for isolates 6 and 18, the greater overall abundance of the unprocessed precursor appears to have exceeded the capacity of SecA to fully translocate it. From this, we hypothesize that when the unprocessed precursor is abundant, SecA which is already hampered in its binding affinity for non-class 1 FHbp, translocates only a portion of this preprolipoprotein to the IM.

### Transcript Level of *fHbp* Influences Sub-Cellular Distribution and Surface Localization of FHbp

**FIGURE 7:**
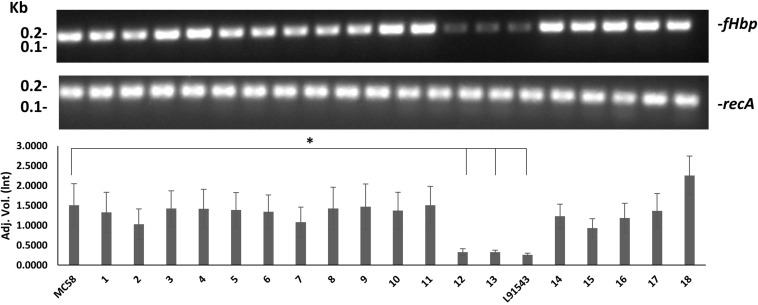
Comparison of *fHbp* transcript level between strains. RT-PCR of *fHbp* and *recA*. One representative experiment of 3 is shown. Band intensity was measured using Image lab v4.0.1 and the data acquired using Linear non-threshold model (lnt). The one-way ANOVA followed by Dunnett’s test was employed for statistical analysis in GraphPad (V. 6). All columns represent mean ± SEM, ^∗^*p* < 0.05 vs. strain MC58.

We speculated that the poor expression of the FHbp preprolipoprotein in L91543 was due to weak transcription of *fHbp*, given that strains are known to vary in their transcription efficiency for *fHbp* (Oriente et al., 2010; Sanders et al., 2012; Cayrou et al., 2018). To test this, RNA was prepared from *A*_600_ 1.0 broth cultures of all 20 strains and RT-PCR performed with invariant *fHbp*-specific primers. L91543 exhibited 5.8-fold reduction in *fHbp* transcript level, and a reduction of 4.6-fold was observed for isolates 12 and 13, compared to MC58 (Figure 7). These three isolates were the same isolates previously found to display FHbp poorly at the cell surface (Figure 5B) hence transcription efficiency influences surface abundance as expected.

**FIGURE 8:**
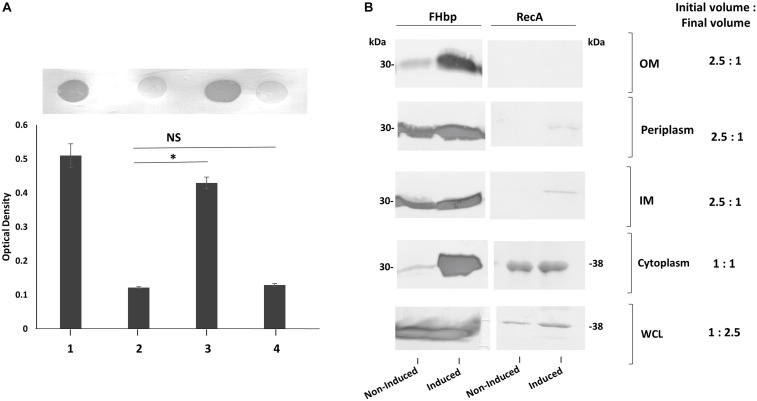
The affect of increasing transcription of *fHbp* on FHbp subcellular localization and surface localization in L91543. **(A)** Immuno-dot blot of whole cells with JAR4. Lanes; 1, MC58, 2, L91543, 3, L91543Δ*fHbp* + L*fHbp* induced with IPTG, 4, L91543Δ*fHbp* + L*fHbp* non-induced. One representative experiment of 5 is shown. The optical density was measured by ImageJ. The ANOVA followed by Dunnett’s test was employed for statistical analysis in GraphPad (V. 6). All columns represent mean ± SEM, ^∗^*p* < 0.05 vs. strain L91543. **(B)** Western immunoblot with JAR4 of different subcellular compartments of strain L91543Δ*fHbp* + L*fHbp* non-induced versus induced. Equal protein loading was confirmed by the determination of RecA protein.

To test our hypothesis that an abundance of non-class 1 FHbp exceeds the capacity for complete translocation across the IM, strong expression of *fHbp* was induced in L91543 and changes in the subcellular distribution of FHbp were investigated. This was made possible by exploiting strain L91543Δ*fHbp* + L*fHbp* wherein this class 2 *fHbp* is under the control of the inducible *lacZ* promoter. First, the amount of surface FHbp preprolipoprotein was compared by immuno-dot blot between isopropyl-β-D-thiogalactopyranoside (IPTG)-induced and non-induced L91543Δ*fHbp* + L*fHbp*, relative to the L91543 parent strain and to MC58. The induced recombinant strain localized unprocessed FHbp to the surface with a similar level to that of MC58 (Figure 8A). In the absence of induction, the preprolipoprotein localized to the periplasm and IM but very poorly to the OM (Figure 8B) similar to that observed for the L91543 parent strain (Figure 8B). Upon induction, not only did the quantity of preprolipoprotein dramatically increase, as expected, but as with isolates 6 and 18, some was retained in the cytoplasm while the remainder localized to the IM, periplasm and OM (Figure 8B). Together the data suggest that a certain threshold level of FHbp in the periplasm is tolerated, as shown for strain L91543 (Figure 6D) and non-induced recombinant strain, L91543Δ*fHbp* + L*fHbp* (Figure 8B). However, over-accumulation in the periplasm appears to force translocation to the OM as observed for induced L91543Δ*fHbp* + L*fHbp* and for isolates 6 and 18 (Figures 6D, 8B).

### Comparison of Biological Activities of Unprocessed and Processed Surface FHbp

The binding of FHbp to fH is vital for the meningococcus to inhibit bacteriolysis by the alternative pathway (Schneider et al., 2006). Crystal structure studies reveal that FHbp folds to form an N-terminal β barrel and a C-terminal β barrel and that the FHbp-fH complex is held together by extensive interactions between both β-barrels and domains 6 and 7 of fH (Schneider et al., 2009). We questioned whether the inclusion of the 26 AA SP at the N terminus of FHbp affects the binding of FHbp to fH. Equivalent cell numbers of each of the 20 isolates were incubated with fH. Anti-fH antibody binding was then measured by Fluorescence-Activated Cell Sorting (FACS). No significant difference in binding of class 1 versus non-class 1 isolates was found.

Since a certain threshold level of FHbp on the surface of cells is required for successful killing by vaccine-induced antibodies (Jiang et al., 2010; McNeil et al., 2018) we questioned whether isolates with non-class 1 FHbp SP that typically display less unprocessed FHbp at the surface may be more resistant to killing by antibodies. First FHbp surface expression was rigorously quantified by FACS analysis with JAR4. Since JAR4 is a monoclonal antibody binding a single epitope (Supplementary Table S1), saturation of meningococcal cells with this antibody should result in the maximal binding of one antibody per FHbp molecule. Commercially available microspheres were used to quantify the number of antibodies bound per cell (ABC) and enable us to infer from this the number of FHbp molecules displayed per cell. The SBA assay was performed using the FHbp-specific monoclonal antibody JAR5 along with JAR4. Cooperativity between these two antibodies is necessary to elicit bactericidal activity in the presence of human complement (Beernink et al., 2008; Welsch et al., 2008; Malito et al., 2016). JAR5 is known to bind the FHbp epitope comprising AA residues 84–91 and 115–123 (Malito et al., 2016) (Supplementary Table S1). Since MC58 and L91543 were previously shown to contrast starkly in their FHbp exposure and susceptibility to killing by FHbp-immunized sera in SBA assays (Newcombe et al., 2014) these strains were used as positive and negative controls respectively to benchmark all other isolates.

**FIGURE 9:**
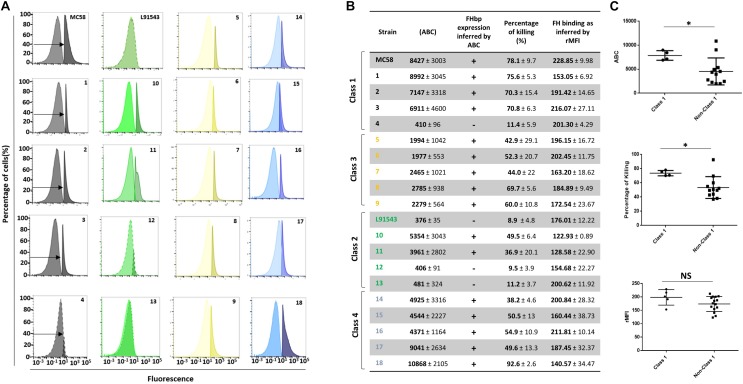
Comparison of biological activities of unprocessed and processed surface-localized FHbp. **(A)** FACS analysis with Mab JAR4. A representative flow cytometry plot for each isolate is shown. The read-outs from negative control samples, cells incubated with secondary antibody only (left peak) were gated (as shown by the arrows). The read-outs from samples incubated with both primary and secondary antibody were overlaid. **(B)** The number of JAR4 antibody molecules bound per cell (ABC) and corresponding prediction for successful killing in SBA assays is denoted by +. The actual percentage killing for each isolate with Mabs JAR4, JAR5 and human complement is shown. The mean ABC for each isolate was derived from three independent FACS experiments and the mean percentage killing derived from four independent SBA experiments, each with two technical replicates. The binding of fH to cells. Values show the Relative Mean of Fluorescence Intensity (rMFI) for total fluorescing cells after incubation with fH and anti-fH PE-conjugated antibody. The values were normalized against the negative control of cells alone incubated with antibody. **(C)** Pooled data for class 1 and non-class 1 isolates, excluding the outliers. Values were analyzed by unpaired *t*-tests with Welsch’s correction. Columns represent mean ± SEM, ^∗^*p* < 0.05 vs. class 1.

We first noted that isolates 4, 12, and 13 displayed very poor surface FHbp at a level comparable with that of L91543 (Figure 9A) supporting our previous immuno-dot blot data for these isolates (Figure 5B). These levels were well below 757 molecules per cell, the threshold level for killing by anti-FHbp antibodies (Biagini et al., 2016). Unsurprisingly, extremely weak bactericidal activity was detected for these isolates in SBA assays with up to 11% killing demonstrated (Figure 9B). Our findings are explained by the poor transcript levels observed for isolates L91543, 12, and 13 (Figure 7) and by failure of JAR4 to bind isolate 4 (Figure 5B). Pooled class 1 isolates were compared with pooled non-class 1 isolates after discounting the four outliers. For ABC, the average was 7869 vs. 4547 molecules (*p* = 0.0038) and for SBA the average was 73.7 vs. 53.4% (*p* = 0.0009) (Figure 9C).

## Discussion

Since the discovery of FHbp as a lipoprotein (Fletcher et al., 2004) it has been assumed that all meningococci that express FHbp synthesize the mature lipoprotein. The failure to process FHbp including lipidation and SP cleavage, in strain L91543 was directly linked to mutations in the SP (L15F) and (T19A) that interrupt Sec-mediated translocation. The correlation between SP mutations and failure to process FHbp was further confirmed in a panel of invasive group B isolates. Remarkably the preprolipoprotein of isolates possessing variant SPs localized to the cell surface but with twofold lower abundance and in the subset of isolates tested, this was attributed to its partial retention in the cytoplasm. For L91543 with poor transcription of *fHbp*, the less abundant preprolipoprotein was all translocated across the IM but none was translocated to the OM for subsequent surface display. This could however be overcome by increasing the transcription strength of *fHbp*.

It is known that Sec-dependent proteins are exported in unfolded states to the Sec-YEG channel (Figure 1) as this channel is only large enough to accommodate unfolded proteins (Li et al., 2016). SecA acts promptly to bind nascent polypeptides as they emerge from the ribosome during translation and maintains the polypeptide in an unfolded state (Huber et al., 2017). Disrupting this interaction causes a partial defect in SecA-mediated translocation (Huber et al., 2017) and reduced efficiency of transport to the IM due to the propensity for the preprolipoprotein to fold and become incompetent for translocation (Steiner et al., 2006). Based on new insights in SecA-mediated translocation (Mao et al., 2016), it is possible that the preprolipoprotein that is translocated is not orientated correctly in the IM such that Lgt fails to recognize this FHbp precursor as substrate.

Our findings suggest that the polar AA, threonine (T), four AAs away from the lipobox of FHbp, is the most critical for correct translocation by SecA in MC58 as the T19A mutation is carried by all non-class 1 isolates (at least 88% of MRF isolates) and is the sole mutation in class 3 isolates (48% of MRF isolates) (Table 1). This supports the work of Vlasuk and co-workers who in the early 80s demonstrated the importance of polar residues close to the lipobox of *E. coli* Lpp, for translocation (Vlasuk et al., 1984). We postulate that in T19A FHbp variants, the reduced efficiency of binding to the SP by SecA results in the propensity for folding with consequent retention of some of this translocation-incompetent preprolipoprotein in the cytoplasm. This retention was evident for both isolates 6 and 18 tested that express an abundance of the preprolipoprotein and for L91543 after induction of strong expression of FHbp (Figures 6D, 8B). The remaining preprolipoprotein was translocated across the IM and some of this was translocated to the OM and further surface-displayed. It is plausible that the SP anchors FHbp to the OM facilitated by its hydrophobic region. Upon accumulation of the unprocessed precursor FHbp in the IM beyond a threshold level, translocation to the OM is forced, probably to prevent clogging which would prohibit the translocation of other OM-destined proteins and be potentially fatal for the cell. We provide data suggesting this translocation is partly mediated by Lnt highlighting a secondary role for Lnt as a chaperone facilitating delivery to Lol. We further show that localization to the surface involves Slam revealing that Slam is not lipoprotein-specific. The broader specificity of Slam, than originally thought, for OM protein localization is plausible given Slam recognizes the C terminal structure of its substrates (Hooda et al., 2016) which may be shared between lipoproteins and OM proteins.

From analysis of the SP sequence of 1895 invasive group B isolates from the United Kingdom MRF collection, as few as 9% are predicted to express FHbp as a lipoprotein. Conversely, at least 88% are predicted to display the unprocessed, precursor at the surface, provided sufficient transcription strength of *fHbp*. A similar prevalence is expected for other isolates circulating around the globe. Given that isolates displaying non-class 1 FHbp SP are far more prevalent than class 1 isolates, there must be a fitness benefit for the former. One explanation for evolutionary selection for the former could be that exportation of unprocessed FHbp is less metabolically costly than employing processing enzymes to modify FHbp prior to localization of the mature lipoprotein to the surface. Alternatively the significantly reduced surface exposure of the preprolipoprotein, shown in this study, may be selected for to reduce anti-FHbp antibody-mediated killing whilst retaining sufficient fH binding. It is worth noting here that we would anticipate seeing even greater differences in SBA responses between Class 1 and non-Class 1 isolates using sera from Trumenba-vaccinated individuals (as opposed to the two Mabs used in this study) since this vaccine specifically targets SP-cleaved, lipidated FHbp (Class 1 FHbp) with polyclonal antibodies generated to multiple epitopes.

To summarize, our results reveal that the AA sequence requirement for lipoprotein SPs to direct translocation to the IM completely and correctly for processing to follow, are more constrained than previously thought. In the DOLOP database of predicted bacterial lipoproteins https://www.mrc-lmb.cam.ac.uk/genomes/dolop/predicted/ab.shtml (Babu et al., 2006) the algorithm does not include the requirement for the polar residue and for many of the proteins in this database, this residue is absent. These proteins are unlikely to undergo maturation to become lipoproteins and thus the size of bacterial lipoproteomes could be over-estimated. More experimental work is required for other bacterial lipoproteins to fully determine the structural features, in particular, critical AAs, that are required for lipoprotein maturation and localization. From this, new programs with greater prediction power can be deployed which would be of importance to scientists developing vaccines based on bacterial lipoproteins, a growing area of research.

Finally, our finding that the FHbp vaccine target is predominantly unprocessed FHbp as opposed to cleaved, lipidated FHbp, is particularly important for Trumenba which is composed exclusively of 2 lipidated variants of FHbp. Altering the vaccine to contain uncleaved, non-lipidated FHbp should improve its target specificity. It is unknown to the public which reference strains are used in Pfizer’s MEASURE assay (Meningococcal Antigen Surface Expression) (Donald et al., 2017) as standards for quantifying FHbp surface abundance on test isolates in order to predict vaccine coverage. With knowledge of the prevalence of 4 SP classes for FHbp – the majority of isolates expressing non-class 1, the choice of reference strains should be re-considered to ensure sufficient representation across these classes. In predicting coverage by Trumenba, knowledge on the nucleotide level of promoter and upstream regulatory sequences (Cayrou et al., 2018) and on the AA level of the SP sequence are both key as the former dictates FHbp expression level and the latter its sub-cellular distribution and importantly surface localization. This sequence information will enable an improved understanding of Trumenba vaccine escape strains and this knowledge can feed into developing improved formulations. Our findings also bear relevance for Bexsero although this vaccine is multivalent and thus less heavily reliant on the successful targeting of FHbp. Our study highlights the importance of multivalent vaccine approaches.

## Data Availability Statement

The FACS data discussed in this publication have been deposited in FlowRepository (Spidlen et al., 2012) and are available through accession numbers: FR-FCM-ZYTU, http://flowrepository.org/id/RvFrLUcAM69ZrTziXZJn622NsvQ gwRuVqoRC28XA9wBxR2KZsAJ2yym5ToAfMt6p, FR-FCM-Z YTV, http://flowrepository.org/id/RvFryhGbJHv5UMqqZ1bEfLE z6aO8QEV0EsQtjKdcSjlMt1rCtFIiT9HmOrMZYBms and FR-FCM-ZYUZ, http://flowrepository.org/id/RvFraaVCkdcJJYpYJO c4CFQNbETeVGwTy1bcNwXpA3YotemW9trGUDNKJFgxXn Ki. The closed genome sequence of L91543 is available under accession number CP016684.

## Author Contributions

RS performed all of the experiments, analyzed the data, and contributed to the experimental design and writing of the manuscript (Materials and Methods and Figures). RG devised the project, supervised the experiments, analyzed the data, and wrote the manuscript. AK closed the genome of L91543. NO and KW assisted with the bioinformatics analyses. CB provided the strains and FHbp protein sequences. AR provided technical advice.

## Conflict of Interest

The authors declare that the research was conducted in the absence of any commercial or financial relationships that could be construed as a potential conflict of interest.

## Supplementary Material

FIGURE S1 Western immunoblotting of WCL of L91543 and L91543Δ*fHbp* with JAR4.

TABLE S1 Multiple alignment of FHbp of isolates used in this study and AA identities across the whole protein relative to MC58. Framed in red and gray boxes are AA residues in the epitopes recognized by JAR4 and JAR5 respectively.

TABLE S2 AA identity of proteins involved in translocation, processing and surface localization of FHbp in L91543 compared to MC58.

## References

[B1] Ames G. F. Prody C. Kustu S. (1984). Simple, rapid, and quantitative release of periplasmic proteins by chloroform. *J. Bacteriol.* 160 1181–1183. 6501229 10.1128/jb.160.3.1181-1183.1984 PMC215841

[B2] Babu M. M. Priya M. L. Selvan A. T. Madera M. Gough J. Aravind L. (2006). A database of bacterial lipoproteins (DOLOP) with functional assignments to predicted lipoproteins. *J. Bacteriol.* 188 2761–2773. 10.1128/jb.188.8.2761-2773.2006 16585737 PMC1446993

[B3] Basta N. E. Christensen H. (2016). 4CMenB vaccine effectiveness: reasons for optimism. *Lancet* 388 2719–2721. 10.1016/s0140-6736(16)32061-x 28100431 PMC5424821

[B4] Beernink P. T. Granoff D. M. (2009). The modular architecture of meningococcal factor H-binding protein. *Microbiology* 155 2873–2883. 10.1099/mic.0.029876-0 19574307 PMC2859308

[B5] Beernink P. T. Shaughnessy J. Braga E. M. Liu Q. Rice P. A. Ram S. (2011). A meningococcal factor H binding protein mutant that eliminates factor H binding enhances protective antibody responses to vaccination. *J. Immunol.* 186 3606–3614. 10.4049/jimmunol.1003470 21325619 PMC3098282

[B6] Beernink P. T. Welsch J. A. Bar-Lev M. Koeberling O. Comanducci M. Granoff D. M. (2008). Fine antigenic specificity and cooperative bactericidal activity of monoclonal antibodies directed at the meningococcal vaccine candidate factor h-binding protein. *Infect. Immun.* 76 4232–4240. 10.1128/IAI.00367-08 18591239 PMC2519416

[B7] Biagini M. Spinsanti M. De Angelis G. Tomei S. Ferlenghi I. Scarselli M. (2016). Expression of factor H binding protein in meningococcal strains can vary at least 15-fold and is genetically determined. *Proc. Natl. Acad. Sci. U.S.A.* 113 2714–2719. 10.1073/pnas.1521142113 26888286 PMC4791009

[B8] Brehony C. Wilson D. J. Maiden M. C. (2009). Variation of the factor H-binding protein of *Neisseria meningitidis*. *Microbiology* 155 4155–4169. 10.1099/mic.0.027995-0 19729409 PMC2801853

[B9] Cayrou C. Akinduko A. A. Mirkes E. M. Lucidarme J. Clark S. A. Green L. R. (2018). Clustered intergenic region sequences as predictors of factor H binding protein expression patterns and for assessing *Neisseria meningitidis* strain coverage by meningococcal vaccines. *PLoS One* 13:e0197186. 10.1371/journal.pone.0197186 29847547 PMC5976157

[B10] Chambonnier G. Roux L. Redelberger D. Fadel F. Filloux A. Sivaneson M. (2016). The hybrid histidine kinase LadS Forms a multicomponent signal transduction system with the GacS/GacA two-component system in *Pseudomonas aeruginosa*. *PLoS Genet.* 12:e1006032. 10.1371/journal.pgen.1006032 27176226 PMC4866733

[B11] Clark V. L. Campbell L. A. Palermo D. A. Evans T. M. Klimpel K. W. (1987). Induction and repression of outer membrane proteins by anaerobic growth of *Neisseria gonorrhoeae*. *Infect. Immun.* 55 1359–1364. 3106220 10.1128/iai.55.6.1359-1364.1987 PMC260520

[B12] da Silva R. A. G. Churchward C. P. Karlyshev A. V. Eleftheriadou O. Snabaitis A. K. Longman M. R. (2017). The role of apolipoprotein N-acyl transferase, Lnt, in the lipidation of factor H binding protein of *Neisseria meningitidis* strain MC58 and its potential as a drug target. *Br. J. Pharmacol.* 174 2247–2260. 10.1111/bph.13660 27784136 PMC5481643

[B13] da Silva R. A. G. Karlyshev A. V. Oldfield N. J. Wooldridge K. G. Bayliss C. D. Ryan A. (2019). Vaccine antigen, Factor H binding protein, is typically a non-lipidated precursor that localises to the meningococcal surface by Slam. *bioRxiv[Pre-Print]*. 10.1101/693374

[B14] Donald R. G. Hawkins J. C. Hao L. Liberator P. Jones T. R. Harris S. L. (2017). Meningococcal serogroup B vaccines: estimating breadth of coverage. *Hum. Vacc. Immunother.* 13 255–265. 10.1080/21645515.2017.1264750 27960595 PMC5328210

[B15] Driessen A. J. Nouwen N. (2008). Protein translocation across the bacterial cytoplasmic membrane. *Annu. Rev. Biochem.* 77 643–667. 10.1146/annurev.biochem.77.061606.160747 18078384

[B16] Fletcher L. D. Bernfield L. Barniak V. Farley J. E. Howell A. Knauf M. (2004). Vaccine potential of the *Neisseria meningitidis* 2086 lipoprotein. *Infect. Immun.* 72 2088–2100. 10.1128/iai.72.4.2088-2100.2004 15039331 PMC375149

[B17] Froger A. Hall J. E. (2007). Transformation of plasmid DNA into *E. coli* using the heat shock method. *J. Vis. Exp.* 6 253. 10.3791/253 18997900 PMC2557105

[B18] Gandhi A. Balmer P. York L. J. (2016). Characteristics of a new meningococcal serogroup B vaccine, bivalent rLP2086 (MenB-FHbp; Trumenba(R)). *Postgrad. Med.* 128 548–556. 10.1080/00325481.2016.1203238 27467048

[B19] Gelis I. Bonvin A. M. Keramisanou D. Koukaki M. Gouridis G. Karamanou S. (2007). Structural basis for signal-sequence recognition by the translocase motor SecA as determined by NMR. *Cell* 131 756–769. 10.1016/j.cell.2007.09.039 18022369 PMC2170882

[B20] Giuntini S. Reason D. C. Granoff D. M. (2011). Complement-mediated bactericidal activity of anti-factor H binding protein monoclonal antibodies against the meningococcus relies upon blocking factor H binding. *Infect. Immun.* 79 3751–3759. 10.1128/IAI.05182-11 21708990 PMC3165461

[B21] Gouridis G. Karamanou S. Gelis I. Kalodimos C. G. Economou A. (2009). Signal peptides are allosteric activators of the protein translocase. *Nature* 462 363–367. 10.1038/nature08559 19924216 PMC2823582

[B22] Hooda Y. Lai C. C.-L. Judd A. Buckwalter C. M. Shin H. E. Gray-Owen S. D. (2016). Slam is an outer membrane protein that is required for the surface display of lipidated virulence factors in *Neisseria*. *Nat. Microbiol.* 1:16009. 10.1038/nmicrobiol.2016.9 27572441

[B23] Horton R. M. (1995). PCR-mediated recombination and mutagenesis. SOEing together tailor-made genes. *Mol. Biotechnol.* 3 93–99. 10.1007/bf02789105 7620981

[B24] Huber D. Jamshad M. Hanmer R. Schibich D. Doring K. Marcomini I. (2017). SecA cotranslationally interacts with nascent substrate proteins in vivo. *J. Bacteriol.* 199: e00622-16. 10.1128/JB.00622-16 27795329 PMC5198489

[B25] Inouye S. Franceschini T. Sato M. Itakura K. Inouye M. (1983). Prolipoprotein signal peptidase of *Escherichia coli* requires a cysteine residue at the cleavage site. *EMBO J.* 2 87–91. 10.1002/j.1460-2075.1983.tb01386.x 11894915 PMC555092

[B26] Jiang H. Q. Hoiseth S. K. Harris S. L. Mcneil L. K. Zhu D. Tan C. (2010). Broad vaccine coverage predicted for a bivalent recombinant factor H binding protein based vaccine to prevent serogroup B meningococcal disease. *Vaccine* 28 6086–6093. 10.1016/j.vaccine.2010.06.083 20619376

[B27] Karlyshev A. V. Snyder L. A. Mcfadden J. Griffin R. (2015). Insight into proteomic investigations of *Neisseria meningitidis* serogroup C strain L91543 from analysis of its genome sequence. *FEMS Microbiol. Lett.* 362:fnv055. 10.1093/femsle/fnv055 25846515

[B28] Kellogg D. S. Jr. Peacock W. L. Jr. Deacon W. E. Brown L. Pirkle D. I. (1963). *Neisseria gonorrhoeae* I. virulence generically linked to clonal variation. *J. Bacteriol.* 85 1274–1279. 14047217 10.1128/jb.85.6.1274-1279.1963 PMC278328

[B29] Kovacs-Simon A. Titball R. W. Michell S. L. (2011). Lipoproteins of bacterial pathogens. *Infect. Immun.* 79 548–561. 10.1128/IAI.00682-10 20974828 PMC3028857

[B30] Li L. Park E. Ling J. Ingram J. Ploegh H. Rapoport T. A. (2016). Crystal structure of a substrate-engaged SecY protein-translocation channel. *Nature* 531 395–399. 10.1038/nature17163 26950603 PMC4855518

[B31] Loh E. Lavender H. Tan F. Tracy A. Tang C. M. (2016). Thermoregulation of Meningococcal fHbp, an important virulence factor and vaccine antigen, is mediated by anti-ribosomal binding site sequences in the open reading frame. *PLoS Pathog.* 12:e1005794. 10.1371/journal.ppat.1005794 27560142 PMC4999090

[B32] Malito E. Lo Surdo P. Veggi D. Santini L. Stefek H. Brunelli B. (2016). *Neisseria meningitidis* factor H-binding protein bound to monoclonal antibody JAR5: implications for antibody synergy. *Biochem. J.* 473 4699–4713. 10.1042/bcj20160806 27784765 PMC6398935

[B33] Mao G. Zhao Y. Kang X. Li Z. Zhang Y. Wang X. (2016). Crystal structure of *E. coli* lipoprotein diacylglyceryl transferase. *Nat. Commun.* 7:10198. 10.1038/ncomms10198 26729647 PMC4728403

[B34] Mascioni A. Moy F. J. Mcneil L. K. Murphy E. Bentley B. E. Camarda R. (2010). NMR dynamics and antibody recognition of the meningococcal lipidated outer membrane protein LP2086 in micellar solution. *Biochim. Biophys. Acta* 1798 87–93. 10.1016/j.bbamem.2009.09.021 19835839

[B35] Masignani V. Comanducci M. Giuliani M. M. Bambini S. Adu-Bobie J. Arico B. (2003). Vaccination against *Neisseria meningitidis* using three variants of the lipoprotein GNA1870. *J. Exp. Med.* 197 789–799. 12642606 10.1084/jem.20021911 PMC2193853

[B36] May K. L. Lehman K. M. Mitchell A. M. Grabowicz M. (2019). A stress response monitoring lipoprotein trafficking to the outer membrane. *mBio* 10:e00618-19. 10.1128/mBio.00618-19 31138744 PMC6538781

[B37] McGuinness B. T. Clarke I. N. Lambden P. R. Barlow A. K. Poolman J. T. Jones D. M. (1991). Point mutation in meningococcal *porA* gene associated with increased endemic disease. *Lancet* 337 514–517. 10.1016/0140-6736(91)91297-8 1705642

[B38] McNeil L. K. Donald R. G. K. Gribenko A. French R. Lambert N. Harris S. L. (2018). Predicting the Susceptibility of meningococcal serogroup B isolates to bactericidal antibodies elicited by bivalent rLP2086, a novel prophylactic vaccine. *mBio* 9:e00036-18. 10.1128/mBio.00036-18 29535195 PMC5850321

[B39] Mehr I. J. Seifert H. S. (1998). Differential roles of homologous recombination pathways in *Neisseria gonorrhoeae* pilin antigenic variation, DNA transformation and DNA repair. *Mol. Microbiol.* 30 697–710. 10.1046/j.1365-2958.1998.01089.x 10094619

[B40] Mori H. Araki M. Hikita C. Tagaya M. Mizushima S. (1997). The hydrophobic region of signal peptides is involved in the interaction with membrane-bound SecA. *Biochim. Biophys. Acta* 1326 23–36. 10.1016/s0005-2736(97)00004-7 9188797

[B41] Newcombe J. Mendum T. A. Ren C. P. Mcfadden J. (2014). Identification of the immunoproteome of the meningococcus by cell surface immunoprecipitation and MS. *Microbiology* 160 429–438. 10.1099/mic.0.071829-0 24275101

[B42] Oliver K. J. Reddin K. M. Bracegirdle P. Hudson M. J. Borrow R. Feavers I. M. (2002). *Neisseria lactamica* protects against experimental meningococcal infection. *Infect. Immun.* 70 3621–3626. 10.1128/iai.70.7.3621-3626.2002 12065503 PMC128090

[B43] Oriente F. Scarlato V. Delany I. (2010). Expression of factor H binding protein of meningococcus responds to oxygen limitation through a dedicated FNR-regulated promoter. *J. Bacteriol.* 192 691–701. 10.1128/JB.01308-09 19948796 PMC2812459

[B44] Ostberg K. L. Derocco A. J. Mistry S. D. Dickinson M. K. Cornelissen C. N. (2013). Conserved regions of gonococcal TbpB are critical for surface exposure and transferrin iron utilization. *Infect. Immun.* 81 3442–3450. 10.1128/IAI.00280-13 23836816 PMC3754215

[B45] Pace D. Pollard A. J. (2007). Meningococcal A, C, Y and W-135 polysaccharide-protein conjugate vaccines. *Arch. Dis. Child* 92 909–915. 10.1136/adc.2006.111500 17895339 PMC2083216

[B46] Rahman M. M. Kolli V. S. Kahler C. M. Shih G. Stephens D. S. Carlson R. W. (2000). The membrane phospholipids of *Neisseria meningitidis* and *Neisseria gonorrhoeae* as characterized by fast atom bombardment mass spectrometry. *Microbiology* 146(Pt 8), 1901–1911. 10.1099/00221287-146-8-1901 10931894

[B47] Rappuoli R. Pizza M. Masignani V. Vadivelu-Pechai K. (2018). Meningococcal B vaccine: the journey from research to real world experience. *Expert Rev. Vacc.* 17 1111–1121. 10.1080/14760584.2018.1547637 30457407

[B48] Sanders H. Brehony C. Maiden M. C. Vipond C. Feavers I. M. (2012). The effect of iron availability on transcription of the *Neisseria meningitidis* fHbp gene varies among clonal complexes. *Microbiology* 158 869–876. 10.1099/mic.0.054957-0 22241045 PMC3949423

[B49] Sankaran K. Wu H. C. (1994). Lipid modification of bacterial prolipoprotein. Transfer of diacylglyceryl moiety from phosphatidylglycerol. *J. Biol. Chem.* 269 19701–19706. 8051048

[B50] Schneider C. A. Rasband W. S. Eliceiri K. W. (2012). NIH Image to ImageJ: 25 years of image analysis. *Nat. Methods* 9 671–675. 10.1038/nmeth.2089 22930834 PMC5554542

[B51] Schneider M. C. Exley R. M. Chan H. Feavers I. Kang Y. H. Sim R. B. (2006). Functional significance of factor H binding to *Neisseria meningitidis*. *J. Immunol.* 176 7566–7575. 10.4049/jimmunol.176.12.7566 16751403

[B52] Schneider M. C. Prosser B. E. Caesar J. J. Kugelberg E. Li S. Zhang Q. (2009). *Neisseria meningitidis* recruits factor H using protein mimicry of host carbohydrates. *Nature* 458 890–893. 10.1038/nature07769 19225461 PMC2670278

[B53] Spidlen J. Breuer K. Rosenberg C. Kotecha N. Brinkman R. R. (2012). FlowRepository: a resource of annotated flow cytometry datasets associated with peer-reviewed publications. *Cytometry A* 81 727–731. 10.1002/cyto.a.22106 22887982

[B54] Steiner D. Forrer P. Stumpp M. T. Pluckthun A. (2006). Signal sequences directing cotranslational translocation expand the range of proteins amenable to phage display. *Nat. Biotechnol.* 24 823–831. 10.1038/nbt1218 16823375

[B55] Tettelin H. Saunders N. J. Heidelberg J. Jeffries A. C. Nelson K. E. Eisen J. A. (2000). Complete genome sequence of *Neisseria meningitidis* serogroup B strain MC58. *Science* 287 1809–1815. 10.1126/science.287.5459.1809 10710307

[B56] Tokuda H. (2009). Biogenesis of outer membranes in Gram-negative bacteria. *Biosci. Biotechnol. Biochem.* 73 465–473. 10.1271/bbb.80778 19270402

[B57] Tokunaga M. Tokunaga H. Wu H. C. (1982). Post-translational modification and processing of *Escherichia coli* prolipoprotein in vitro. *Proc. Natl. Acad. Sci. U.S.A.* 79 2255–2259. 10.1073/pnas.79.7.2255 7048314 PMC346170

[B58] Vernikos G. Medini D. (2014). Bexsero(R) chronicle. *Pathog. Glob. Health* 108 305–316. 10.1179/2047773214Y.0000000162 25417906 PMC4241781

[B59] Vlasuk G. P. Inouye S. Inouye M. (1984). Effects of replacing serine and threonine residues within the signal peptide on the secretion of the major outer membrane lipoprotein of *Escherichia coli*. *J. Biol. Chem.* 259 6195–6200. 6427210

[B60] Vogeley L. El Arnaout T. Bailey J. Stansfeld P. J. Boland C. Caffrey M. (2016). Structural basis of lipoprotein signal peptidase II action and inhibition by the antibiotic globomycin. *Science* 351 876–880. 10.1126/science.aad3747 26912896

[B61] Vu D. M. Pajon R. Reason D. C. Granoff D. M. (2012). A broadly cross-reactive monoclonal antibody against an epitope on the n-terminus of meningococcal fHbp. *Sci. Rep.* 2:341. 10.1038/srep00341 22461972 PMC3314305

[B62] Welsch J. A. Ram S. Koeberling O. Granoff D. M. (2008). Complement-dependent synergistic bactericidal activity of antibodies against factor H-binding protein, a sparsely distributed meningococcal vaccine antigen. *J. Infect. Dis.* 197 1053–1061. 10.1086/528994 18419542

[B63] Welsch J. A. Rossi R. Comanducci M. Granoff D. M. (2004). Protective activity of monoclonal antibodies to genome-derived neisserial antigen 1870, a *Neisseria meningitidis* candidate vaccine. *J. Immunol.* 172 5606–5615. 10.4049/jimmunol.172.9.5606 15100304

[B64] Zhang Q. Li Y. Tang C. M. (2010). The role of the exopolyphosphatase PPX in avoidance by *Neisseria meningitidis* of complement-mediated killing. *J. Biol. Chem.* 285 34259–34268. 10.1074/jbc.M110.154393 20736171 PMC2962524

[B65] Zlotnick G. W. Jones T. R. Liberator P. Hao L. Harris S. Mcneil L. K. (2015). The discovery and development of a novel vaccine to protect against *Neisseria meningitidis* Serogroup B Disease. *Hum. Vacc. Immunother.* 11 5–13. 10.4161/hv.34293 25483509 PMC4514153

