# Corrigendum: Variant Signal Peptides of Vaccine Antigen, FHbp, Impair Processing Affecting Surface Localization and Antibody-Mediated Killing in Most Meningococcal Isolates

**DOI:** 10.3389/fmicb.2020.00055

**Published:** 2020-02-05

**Authors:** Ronni A. G. da Silva, Andrey V. Karlyshev, Neil J. Oldfield, Karl G. Wooldridge, Christopher D. Bayliss, Ali Ryan, Ruth Griffin

**Affiliations:** ^1^Centre for Biomolecular Sciences, University of Nottingham, Nottingham, United Kingdom; ^2^School of Life Sciences, Pharmacy and Chemistry, Kingston University, Kingston upon Thames, United Kingdom; ^3^Department of Genetics and Genome Biology, University of Leicester, Leicester, United Kingdom

**Keywords:** meningoccocus, FHbp, vaccine, signal peptide, lipoprotein, Lnt, Slam

In the original article, the labelling of “Class 2” and “Class 3” isolates was switched erroneously in Table 2 and Figure 9B. The corrected Table 2 and Figure 9 appear below.

**Table 2:** MenB invasive isolates used in this study.

**Class**	**Isolate number**	**Isolate, full name**
1	–	MC58
	1	H44/76
	2	M10_240684
	3	M10_240701
	4	M02_241729
3	5	M10_240579
	6	M13_240525
	7	M04_241215
	8	M11_241066
	9	M13_240614
2	–	L91543
	10	M10_240750
	11	M13_240675
	12	M11_240236
	13	M12_240006
4	14	M11_241033
	15	M14_240367
	16	M02_240210
	17	M11_240077
	18	M13_240486

*The SP class is indicated for each isolate*.

**Figure 9:**
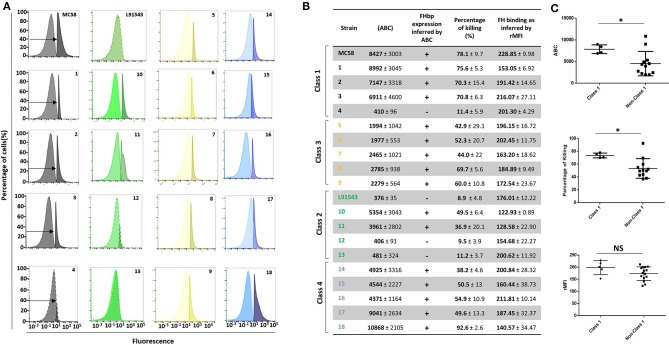
Comparison of biological activities of unprocessed and processed surface-localized FHbp. **(A)** FACS analysis with Mab JAR4. A representative flow cytometry plot for each isolate is shown. The read-outs from negative control samples, cells incubated with secondary antibody only (left peak) were gated (as shown by the arrows). The read-outs from samples incubated with both primary and secondary antibody were overlaid. **(B)** The number of JAR4 antibody molecules bound per cell (ABC) and corresponding prediction for successful killing in SBA assays is denoted by +. The actual percentage killing for each isolate with Mabs JAR4, JAR5 and human complement is shown. The mean ABC for each isolate was derived from three independent FACS experiments and the mean percentage killing derived from four independent SBA experiments, each with two technical replicates. The binding of fH to cells. Values show the Relative Mean of Fluorescence Intensity (rMFI) for total fluorescing cells after incubation with fH and anti-fH PE-conjugated antibody. The values were normalized against the negative control of cells alone incubated with antibody. **(C)** Pooled data for class 1 and non-class 1 isolates, excluding the outliers. Values were analyzed by unpaired *t*-tests with Welsch's correction. Columns represent mean ± SEM, *p < 0.05 vs. class 1.

Further, the year the Trumenba vaccine was licensed is incorrectly provided as “2015” and should be “2014”.

A correction has been made to the **Introduction**, paragraph three.

“Through an accelerated approval process, both Trumemba (Pfizer) and Bexsero (GSK) were licensed by the FDA in 2014 and 2015 respectively for immunization to prevent invasive disease by meningococcal group B in the United States in individuals 10 to 25 years of age. Trumenba comprises two recombinant FHbps, one from subfamily A, the other from subfamily B, both containing the lipid moiety found in the native protein (Fletcher et al., 2004; Gandhi et al., 2016). A recombinant non-lipidated form of FHbp from subfamily B is also one of the antigens of the Bexsero vaccine (GSK) (Vernikos and Medini, 2014) licensed for infants from 2 months of age in Europe in 2013 and, like Trumenba, now licensed globally (Basta and Christensen, 2016).”

The authors apologize for these errors and state that this does not change the scientific conclusions of the article in any way. The original article has been updated.

