# Supplementary figures and images for: Variant Signal Peptides of Vaccine Antigen, FHbp, Impair Processing Affecting Surface Localization and Antibody-Mediated Killing in Most Meningococcal Isolates
Source: Front Microbiol. 2019 Dec 19;10:2847. doi: 10.3389/fmicb.2019.02847 (PMC6930937; doi:10.3389/fmicb.2019.02847)

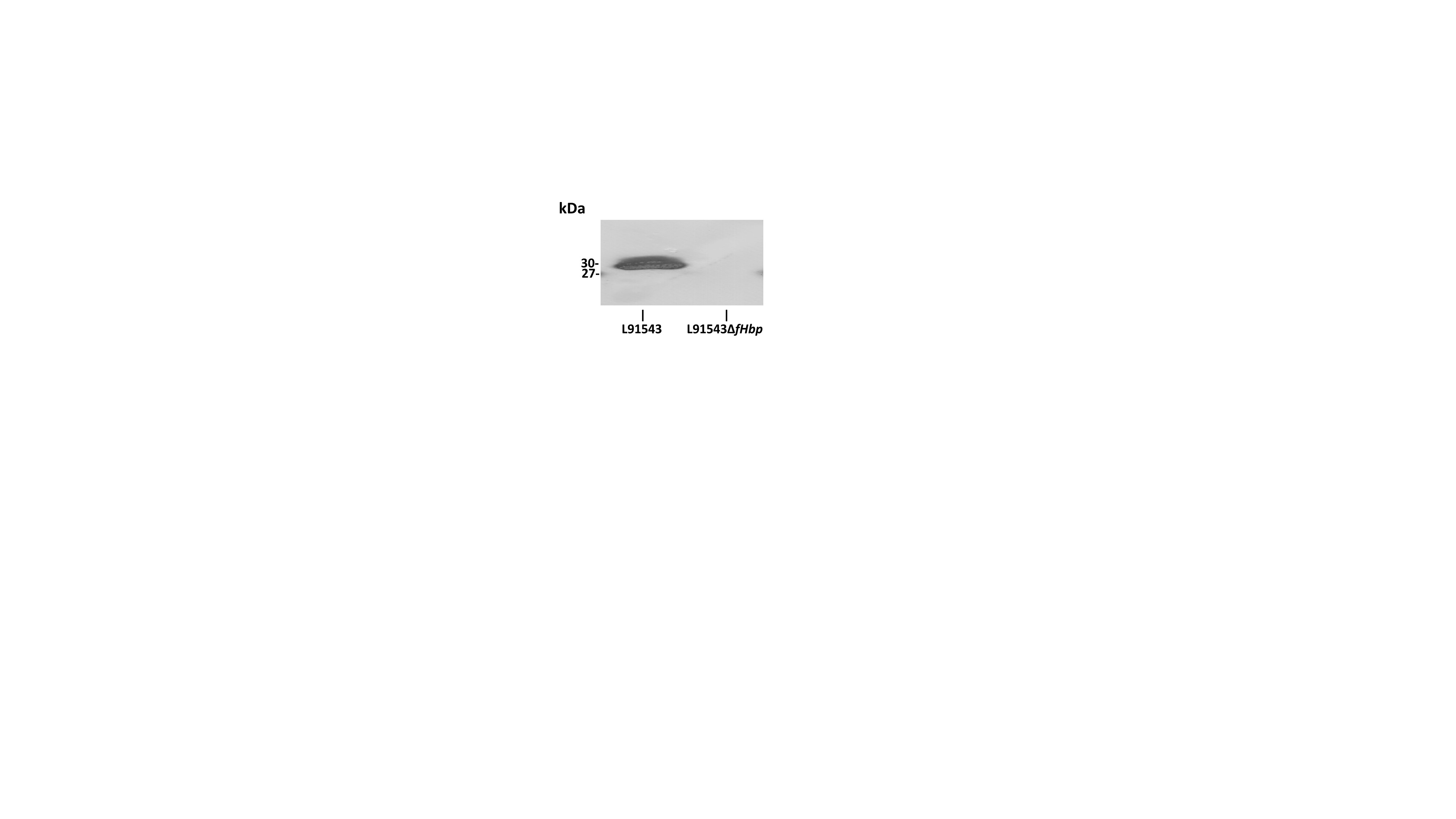

Supplement: FIGURE S1 — Western immunoblotting of WCL of L91543 and L91543ΔfHbp with JAR4. [file Image_1.JPEG]
