# Supplementary material for: Variant Signal Peptides of Vaccine Antigen, FHbp, Impair Processing Affecting Surface Localization and Antibody-Mediated Killing in Most Meningococcal Isolates
Source: Front Microbiol. 2019 Dec 19;10:2847. doi: 10.3389/fmicb.2019.02847 (PMC6930937; doi:10.3389/fmicb.2019.02847)
Supplement: TABLE S1 — Multiple alignment of FHbp of isolates used in this study and AA identities across the whole protein relative to MC58. Framed in red and gray boxes are AA residues in the epitopes recognized by JAR4 and JAR5 respectively. [file Table_1.pdf]

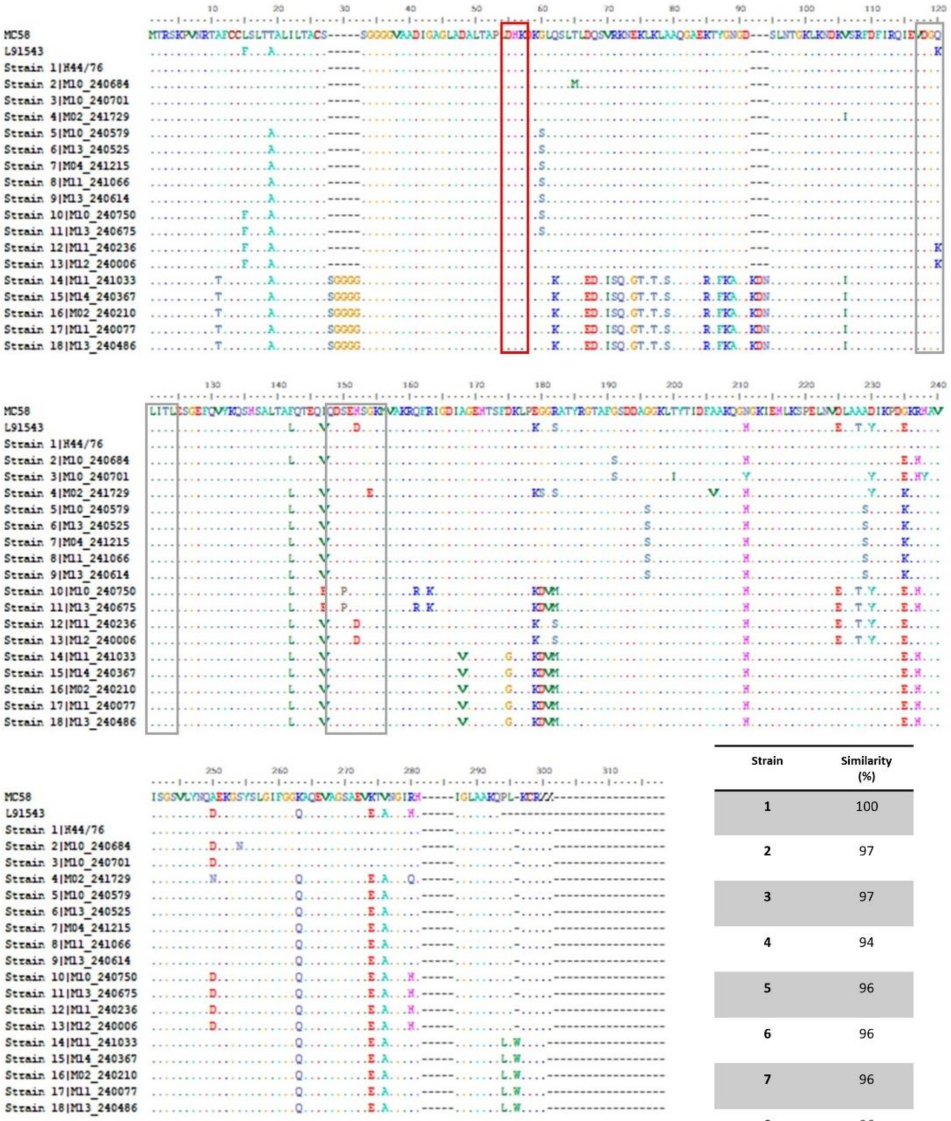

Strain	Similarity (%)
1	100
2	97
3	97
4	94
5	96
6	96
7	96
8	96
9	96
L91543	93
10	92
11	92
12	94
13	94
14	86
15	86
16	86
17	86
18	86
