# Supplementary material for: Variant Signal Peptides of Vaccine Antigen, FHbp, Impair Processing Affecting Surface Localization and Antibody-Mediated Killing in Most Meningococcal Isolates
Source: Front Microbiol. 2019 Dec 19;10:2847. doi: 10.3389/fmicb.2019.02847 (PMC6930937; doi:10.3389/fmicb.2019.02847)
Supplement: TABLE S2 — AA identity of proteins involved in translocation, processing and surface localization of FHbp in L91543 compared to MC58. [file Table_2.docx]

**Table S2** Amino acid identity of proteins involved in translocation, processing and surface localisation of FHbp in L91543 compared to MC58 (da Silva et al., 2017)

| MC58 (Accession Number: NC_003112) | | | L91543 (Accession Number: CP016684) | | | |
| --- | --- | --- | --- | --- | --- | --- |
| Protein | **ID** | **Gene** | **ID** |  | **AA Identity (%)** |  |
| FHbp | NC_003112.2/  NMB1870 | *fhbp* | ANW71006.1 |  | 93 |  |
| PROCESSING Machinery | | | | | | |
| Lnt | NP_273755.1/ NMB0713 | *lnt* | ANW71313.1 |  | 99 |  |
| LspA | NP_274829.1/ NMB1832 | *lspA* | ANW71045.1 |  | 100 |  |
| Lgt | NP_274105.1/ NMB1072 | *lgt* | ANW71667.1 |  | 98 |  |
| SORTING MACHINERY | | | | | | |
| LolA | NP_273666.1/ NMB0622 | *lolA* | ANW71222.1 |  | 99 |  |
| LolB | NP_273914.1/ NMB0873 | *lolB* | ANW71457.1 |  | 99 |  |
| LolF | NP_274259.1/ NMB1235 | *lolF* | ANW71760.1 |  | 99 |  |
| LolD | NP_274258.1/ NMB1234 | *lolD* | ANW71759.1 |  | 97 |  |
| Slam | NP_273362.1/ NMB0313 | *Slam* | ANW70408.1 |  | 99 |  |
| SEC TRANSLOCON | | | | | | |
| SecA | NP_274543.1/ NMB1536 | *secA* | ANW70027.1 |  | 99 |  |
| SecB | NP_274788.1/ NMB1789 | *secB* | ANW71093.1 |  | 100 |  |
| SecD | NP_273651.1/ NMB0607 | *secD* | ANW71209.1 |  | 99 |  |
| SecE | NP_273183.1/ NMB0125 | *secE* | ANW70781.1 |  | 100 |  |
| SecG | NP_274884.1/ NMB1888 | *secG* | ANW70989.1 |  | 99 |  |
| SecF | NP_273652.1/ NMB0608 | *secF* | ANW71210.1 |  | 99 |  |
| SecY | NP_273220.1/ NMB0162 | *secY* | ANW70814.1 |  | 100 |  |
